# Expanding the genetic engineering toolbox for the metabolically flexible acetogen *Eubacterium limosum*

**DOI:** 10.1093/jimb/kuac019

**Published:** 2022-07-26

**Authors:** Patrick A Sanford, Benjamin M Woolston

**Affiliations:** Department of Chemical Engineering, Northeastern University, 360 Huntington Avenue, 223 Cullinane, Boston, MA 02115, USA; Department of Chemical Engineering, Northeastern University, 360 Huntington Avenue, 223 Cullinane, Boston, MA 02115, USA

**Keywords:** non-model, C1 fermentation, synthetic biology

## Abstract

Acetogenic bacteria are an increasingly popular choice for producing fuels and chemicals from single carbon (C1) substrates. *Eubacterium limosum* is a promising acetogen with several native advantages, including the ability to catabolize a wide repertoire of C1 feedstocks and the ability to grow well on agar plates. However, despite its promise as a strain for synthetic biology and metabolic engineering, there are insufficient engineering tools and molecular biology knowledge to leverage its native strengths for these applications. To capitalize on the natural advantages of this organism, here we extended its limited engineering toolbox. We evaluated the copy number of three common plasmid origins of replication and devised a method of controlling copy number and heterologous gene expression level by modulating antibiotic concentration. We further quantitatively assessed the strength and regulatory tightness of a panel of promoters, developing a series of well-characterized vectors for gene expression at varying levels. In addition, we developed a black/white colorimetric genetic reporter assay and leveraged the high oxygen tolerance of *E. limosum* to develop a simple and rapid transformation protocol that enables benchtop transformation. Finally, we developed two new antibiotic selection markers—doubling the number available for this organism. These developments will enable enhanced metabolic engineering and synthetic biology work with *E. limosum*.

## Introduction

Acetogenic bacteria, defined as bacteria which encode the reductive acetyl-CoA, or Wood–Ljungdahl pathway (WLP), and produce acetate as a primary byproduct, have recently gained interest as organisms of industrial applicability due to their ability to consume single carbon (C1) substrates such as carbon dioxide (Drake et al., 1997; Jin et al., 2020; Müller, 2019; Venkata Mohan et al., 2016). Single carbon substrates as a feedstock for fermentation are attractive due to their abundance, non-competition with food sources, and established industrial infrastructure. Amongst acetogens, the bacterium *Eubacterium limosum* has gained increasing prominence thanks to its metabolic flexibility and role as a prominent member of the human gut microbiome (Mukherjee et al., 2020). Like most other acetogens, it can utilize carbon monoxide and carbon dioxide, but in addition to this, it possesses the ability to directly metabolize formate and methanol into products with high efficiency. This is increasingly important as the concept of circular bio-economies based on methanol and formate has gained significant traction in recent years; these substrates are non-gaseous, making them easier to handle and transport and eliminating gas-liquid mass transfer limitations during fermentation (Cotton et al., 2020; Schrader et al., 2009; Yishai et al., 2016). This flexibility makes *E. limosum* a particularly attractive host for industrial production of value-added products via metabolic engineering (Flaiz et al., 2021; Kremp & Müller, 2021; Loubiere et al., 1992; Sanford & Woolston, 2022). Interest in *E. limosum* has therefore been increasing in recent years, leading to research developments in its engineering tractability, including an engineered strain that produces acetone, the implementation of a CRISPR-Cas9 system, the sequencing of numerous genomes, and elucidation of native regulatory machinery (Flaiz et al., 2021; Kelly et al., 2016; Roh et al., 2011; Shin et al., 2019; Song et al., 2022).

**Fig. 1.**
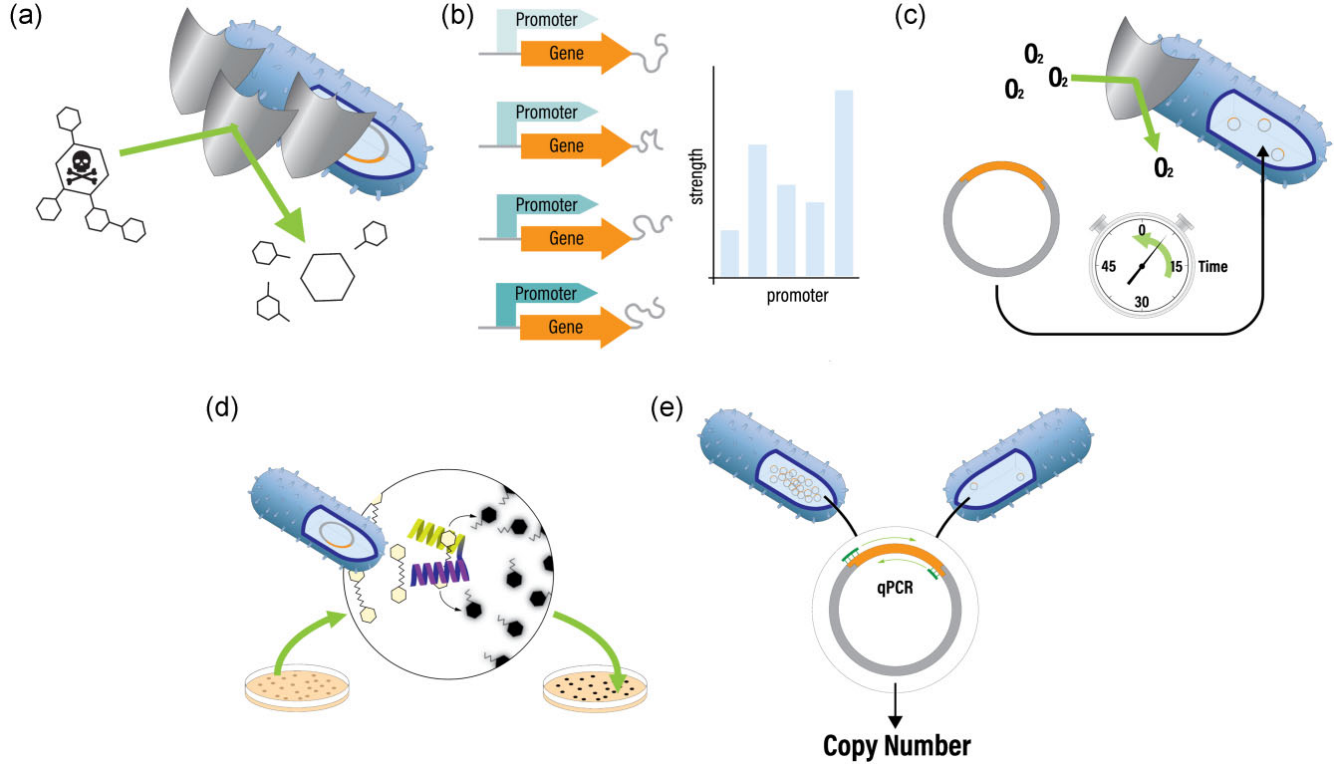
New additions to the engineering toolbox for *E. limosum*: (a) Development of new antibiotic selection markers, (b) Quantitative assessment of promoter strength, (c) Transformation protocol development with determination of the extent of native oxygen tolerance (d) development of colorimetric reporter assay for gene expression, and (e) determination of copy number of origins of replication in plasmids commonly used in acetogens.

Despite recent progress, this organism lacks sufficient engineering tools to fully leverage its native strengths for application in metabolic engineering (Jin et al., 2020). Efforts to develop these engineering tools have also been hindered since, as an acetogen encoding the WLP, it is a strict anaerobe by nature (Schuchmann and Müller, 2014). Here, we present a foundational engineering toolbox for *E. limosum* that both makes the organism easier to work with and, for the first time, demonstrates aerobic engineering procedures. We developed a colorimetric genetic screening assay, assessed oxygen tolerance for bench work and transformation outside of anoxic conditions, determined the copy number of common acetogenic plasmid origins of replication, and quantitatively assessed the strength of a panel of inducible and constitutive promoters commonly used in acetogen metabolic engineering, and report the discovery of new antibiotic selection markers (Fig. 1). These additions to the existing engineering tools should significantly speed the development of *E. limosum* as an industrially relevant engineering host for the conversion of C1 substrates into value-added products and enable metabolic engineering to leverage its superior native metabolic capabilities.

## Materials/methods

### Reagents, bacterial strains, and growth conditions

All plasmid propagation and construction were done using *E. coli* NEB5α from New England BioLabs (NEB). Growth of *E. coli* was done at 37°C exclusively on aerobic LB media or LB agar media supplemented with either 50 μg/ml chloramphenicol or 250 μg/ml erythromycin. All enzymes were acquired from NEB, all DNA synthesis was carried out by Integrated DNA Technologies (IDT), and all oligos were obtained from GeneWiz/Azenta. All chemical reagents were obtained from Sigma-Adrich unless otherwise specified. Miniprep and PCR cleanup kits were obtained from Zymo Research, and gDNA prep and gel purification kits were obtained from NEB. Unless otherwise stated, all enzyme digests were carried out at 37°C for 1 h. All work in *E. limosum* was performed in strain ATCC 8486 obtained from DSMZ. Unless otherwise specified, all steps involving *E. limosum* were executed in an Anaerobe Systems AS-580 anaerobic chamber with a 10% CO_2_, 5% H_2_, and 85% N_2_ gas blend. *Eubacterium limosum* was cultivated at 37°C without agitation. Liquid culture was performed with DSMZ 135 medium without sulfide, and solid culture on reinforced clostridial medium (RCM) agar plates at an agar concentration of 2%.

### Routine transformation

*Eubacterium limosum* from cryostock was inoculated into 50 ml of DSMZ 135 medium in an anoxic 50 ml centrifuge tube with a cap containing an o-ring using a pipette tip stab and grown up for 18 h until reaching an early exponential phase (OD_600_ 0.3–0.5). The tube was chilled on ice for 20 min and then removed from the chamber and centrifuged at 15 000 g for 10 min at 4°C, returned to the chamber, and the supernatant decanted. Cells were fully resuspended in 10 ml of ice-cold anoxic 270 mM sucrose, removed from the chamber and spun at 15 000 g for 10 min at 4°C. Cells were returned to the chamber, the supernatant was discarded, and cells were resuspended fully in anoxic ice cold 20% glycerol(v/v)/270 mM sucrose buffer and centrifuged under the same conditions again. The supernatant was discarded, and the cells were resuspended in the leftover fluid (∼500 μl). Competent cells were aliquoted in 50 μl portions into anoxic ice-cold microcentrifuge tubes and stored at –80°C. Routine transformations were performed by thawing competent cells on ice and adding 0.1–2 μg of DNA in ≤2 μl, flicking to mix, and incubating on ice for 3 min. Cells were transferred to an ice cold 2 mm gap-width Bio-Rad electroporation cuvette, tapped to settle, and shocked at 2500 v, 600 Ω, and 25 uF, exponential decay protocol in a Bio-Rad GenePulser XCell. A total of 950 μl of room temperature DSMZ 135 media was added to the cuvette immediately following the pulse and pipetted gently up and down several times to mix. Cuvettes were then placed in a 37°C incubator without agitation for 6 h to recover before plating 200 μl of undiluted cells.

### Plasmid copy number

Plasmids pCL2, pMTL82254, and pMTL83151 were each individually transformed into *E. limosum* ATCC 8486 and colonies were selected on RCM agar plates with the appropriate antibiotic (50 μg/ml thiamphenicol for pCL2 and pMTL83151 and 5 μg/ml clarithromycin for pMTL82254). After overnight growth on a plate, half of one colony from each was picked into 50 μl molecular biology grade water in a PCR tube and subjected to the following lysis treatment: Cells were fully resuspended/mixed by pipetting; the mixture was incubated at 98°C for 10 min and then frozen at –20°C for 20 min. These mixtures were used as the template for the agar plate stage qPCR reactions. The other half of each colony was used to inoculate a 5 ml culture for each strain supplemented with the appropriate antibiotic. Tubes were placed in a 37°C incubator in the anaerobic chamber. A 50 μl sample of each culture was taken from each tube after 16 h of growth and subjected to the same lysis treatment and used for the early liquid stage qPCR reaction. After a total of 66 h of growth, a 10 μl sample of each culture was taken and mixed with 40 μl of molecular biology grade water to dilute. They were then subjected to the same lysis procedure and used for the late-stage liquid qPCR reactions. All cultures were prepared in biological triplicate.

Plasmid copy number was determined using qPCR with a protocol modified from one described previously in *E. coli* (Lee et al., 2006). The reaction was performed in the same way for each sample across the three time points. Each reaction was run with 8× technical replicates. For each sample, two sets of 8× replicates were run. The first set with primers specific for gDNA from *E. limosum* (oAS45 and oAS46) and the second set with primers specific for the plasmid (oPAS95 and oPAS96). All plasmids contained the same binding sequences for the oligos, so qPCR reactions were identical across plasmids. Oligo binding locations in the gDNA were assessed with BLAST to ensure that they were unique. The gDNA amplicon was 83 bases and the plasmid amplicon was 82 bases. qPCR reactions were assembled on ice with 10 μl of Luna 2× qPCR dye from NEB, 0.5 μl of each 10 μM oligo, 1 μl lysate, and 8 μl molecular biology grade water. Reactions were carried out in a BioRad CFX96 Real-Time System C1000 Thermal Cycler with the following protocol: 95°C (1 m)–95°C (15 sec)–60°C (30 sec) + plate read with 60 cycles. The copy number was calculated by comparing the Cq of amplification in gDNA vs. plasmid. There is only one copy of the genome region amplified; therefore, the total number of copies of DNA needed to reach the Cq can be calculated and used to back-calculate the initial number of plasmids per cell. This is done in conjunction with *E*, the PCR amplification efficiency, calculated from a standard curve:

(1) \begin{equation*}gDNA{\rm{\ }}copies{\rm{\ }}at{\rm{\ }}Cq{\rm{\ }} \!\!=\!\! {\rm{\ }}{\left( {1 + E} \right)}^{Cq{\rm{\ }}\left( {gDNA} \right)},\end{equation*}

(2) \begin{equation*}Plasmid{\rm{\ }}copy{\rm{\ }}number{\rm{\ }} \!\!=\!\! {\rm{\ }}\frac{{{{\left( {1 + {E}_{gDNA}} \right)}}^{Cq\left( {gDNA} \right)}}}{{{{\left( {1 + {E}_{plasmid}} \right)}}^{Cq\left( {plasmid} \right)}}}.\end{equation*}

A standard curve was generated for each qPCR reaction to calculate E: the PCR amplification efficiency. Pure plasmid was isolated with a plasmid miniprep kit and pure gDNA was isolated with a gDNA extraction kit. qPCR reactions were run on each template in duplicate 10-fold serial dilutions from 1 × 10^7^ copies/rxn to 1 × 10^3^ copies/rxn. The Cq values were plotted on a log scale against the initial number of template copies. The *E*-value was then calculated with the following equation: $E\ = {10}^{\frac{{ - 1}}{{slope}}}\ - 1$. All plasmid qPCR amplifications were found to have an *E*-value of 1, and gDNA amplification an *E*-value of ∼0.843. The octuplicate Cq values for each qPCR reaction were averaged before the calculation of plasmid copy number.

In order to compare the difference selection marker makes on copy number across plasmids with the same ori, we constructed plasmid pCL2.1 based on the original pCL2 vector. This was done by digesting pCL2 with AfeI and NheI to excise the *catP* cassette and purifying the backbone via gel purification. Then, the *ermB* cassette was PCR amplified from pMTL82254 with flanking AfeI and NheI cut sites, digested with these enzymes, and purified using a PCR cleanup kit. The resultant fragments were assembled via T4 DNA ligase to create pCL2.1.

### Colorimetric reporter assay

The *BgaB* gene was codon harmonized for *E. limosum* using the Galaxy project codon harmonizer and synthesized (Claassens et al., 2017). Plasmid pELIM2.2 was constructed by Gibson Assembly of pCL2 digested with MfeI and XmnI to isolate the pIP404 origin of replication and PCR products of the *E. coli* origin of replication, *catP* gene from pCL2, and the strong constitutive *E. limosum* nifJp promoter artificially synthesized. The assembled plasmid was stored in *E. coli*, miniprepped, and transformed into *E. limosum*, which was maintained on RCM agar plates supplemented with 50 μg/ml of thiamphenicol. Plating tests were done by growing up 5 ml overnight cultures, diluting 1:100 000 in DSMZ 135 media, and spreading 200 μl onto plates using plating beads. S-Gal plates were prepared with 400 μg/ml ammonium ferric sulfate and 250 μg/ml S-Gal. Induction of liquid cultures was done by adding the same concentrations of each reagent from 10 mg/ml liquid stock solutions in water and dimethyl sulfoxide (DMSO), respectively.

### Oxygen resistance

Test transformations for protocol development were performed in triplicate in the same general fashion as described above. Three different shock conditions were tested, each with ∼1 μg of pCL2 DNA: (1) 1 mm gap-width cuvette, 625, 600Ω, 25μF, and exponential decay protocol. (2) 2000 v, 600 Ω, 25 μF, and exponential decay protocol. (3) 2500 v, 600 Ω, 25 μF, and exponential decay protocol. Following recovery, 200 μl of cells were plated directly onto an RCM agar plate containing 50 μg/ml thiamphenicol. For each transformation. a 1:10 000 dilution was prepared in DSMZ 135 and 200 μl plated onto an RCM agar plate without antibiotics to compare with the selection plate and assess overall transformation efficiency. Plates were grown anoxically at 37°C for at least 3 days before colonies were counted. Following determination of optimal shock conditions (2500 v, 600 Ω, and 25 μF), triplicate transformations were done with a dilution series of pCL2 plasmid DNA from 2 μg to 0.1 μg (2, 1.5, 1, 0.75, 0.5, 0.4, 0.2, 0.1) to assess optimal DNA mass for transformation. Oxygen tolerance for transformations was assessed using the optimized competent cell and transformation protocol. Transformations were done with 400 ng of pCL2 plasmid DNA.

A 5 ml culture of *E. limosum* was grown anoxically overnight in DSMZ 135 media at 37°C. Inside the anaerobic chamber, we prepared 30× sterile microcentrifuge tubes (10× sets of triplicates) with 990 μl sterile anoxic phosphate buffered saline (PBS) and 10 μl of liquid culture aliquoted into each tube. Tubes were mixed well by pipetting. This dilution was done to break up any extracellular substance formation, which may give cells an advantage in protecting themselves from oxygen. Nine sets of tubes were removed from the anerobic chamber and placed on the benchtop in atmospheric conditions. The tubes were then opened, and the rack tented with sterile aluminum foil to maintain sterility. One set of tubes was returned to the anaerobic chamber at each time interval: 30 sec, 10 min, 20 min, 30 min, 45 min, 1 h, 1.5 h, 2 h, and 4 h. After the last set had been returned to the chamber, each tube was well mixed and an overall 1:100 000 dilution in PBS was prepared for each. A total of 100 μl from each 1:100 000 dilution was plated onto RCM agar plates using plating beads. Plates were placed in a 37°C incubator in the anaerobic chamber to grow up for 3 days. Colonies were then counted, and cell viability was assessed.

### Implementation of new selectable markers

First, antibiotics were tested by preparing separate RCM agar plates with 0.5, 1, 5, 10, 15, 20, 25, 35, and 50 μg/ml tetracycline, ampicillin, carbenicillin, and spectinomycin. Wild-type *E. limosum* was grown overnight in liquid culture and patched onto each plate using an inoculation loop. Plates were incubated at 37°C and inspected visually after 3 days for colony formation. Next, the *ampR* gene conferring resistance against ampicillin and carbenicillin was PCR amplified from pCT5-bac1.8 using oligos oPAS116 and oPAS113, the *tet* genes conferring resistance against tetracycline and its derivates were PCR amplified from synthesized DNA using oligos oPAS110 and oPAS117 for *tet(L)*, oPAS130 and oPAS131 for *tet(M)*, and oPAS132 and oPAS133 for *tet(W)*, and finally, *ermB* conferring resistance to clarithromycin (as a positive control) amplified from pMTL82254 with oligos oPAS064 and oPAS065. All products contained NruI and BsrGI cut sites on the 5ʹ and 3ʹ ends, respectively, and were digested and purified with DNA cleanup kit.

Plasmid pVpas3 was created by digesting pCL2 with PvuI and SapI and gel purifying the backbone fragment. A new cloning site block encoding the nifJp promoter, three multiple cloning sites, ribosome binding sites, and the rrnBt terminator was synthesized with PvuI and SapI cut sites on the 5ʹ and 3ʹ ends, respectively. The synthesized DNA was amplified by PCR with oligos oPAS99 and oPAS100 and digested with PvuI and SapI. pVpas3 was generated by T4 ligation of these two linear products. The plasmid was then digested with NruI and BsrGI and purified by gel purification. Plasmids pELerm, pELtetR2, pELtetW, pELtetM, and pELampR2 were created by T4 DNA ligase assembly between digested vector and digested *ermB, tet(L), tet(W), tet(M)*, and *ampR* PCR products, respectively. Plasmids were stored in *E. coli*, miniprepped, and transformed into wild-type *E. limosum*. Following recovery, 200 μl of cells were plated onto RCM agar plates containing a titration series of 0.5, 1, 5, 10, 15, 20, 25, 35, and 50 μg/ml of their respective antibiotic (clarithromycin for *ermB*, ampicillin for *ampR*, carbenicillin for *ampR*, and tetracycline for *tet*). A 1:10 000 dilution in DSMZ 135 media was also prepared from each transformation and 200 μl plated onto RCM plates without antibiotics. Plates were grown anoxically at 37°C for 3 days before colonies were counted. Successful colonies were then propagated in liquid culture in DSMZ 135 media with 5 μg/ml of the appropriate antibiotic. Inhibition titrations were performed by bisecting selective plates at concentrations of 0.5, 1, 5, 10, 15, 20, 25, 35, and 50 μg/ml and spotting out 5 μl of wild type liquid culture and resistant engineered culture onto half of each plate. The strength of expression was determined qualitatively by comparing patch size and strength between engineered and wild-type strains on selective plates.

Finally, vector plasmids pVEL1, pVEL2, and pVEL4 were generated from the pVpas3 plasmid with the antibiotic resistance markers *catP, ermB*, and *Tet(M)*, respectively. Plasmid pVEL1 was constructed by digesting pVpas3 with two blunt cutters, FspI and NruI, to remove the nifJp promoter and then re-ligated with T4 DNA ligase. pVEL2 was constructed by digesting pVEL1 with AfeI and NheI to remove *catP*. Then, *ermB* with regulatory elements was PCR amplified from pMTL82254 with oligos oPAS065 and oPAS066, digested with AfeI and NheI, and assembled into digested pVEL1 by Gibson Assembly. Plasmid pVEL4 was assembled by first digesting pVEL1 with AfeI and NheI to remove *catP*. Next, *aadA* (spectinomycin resistance) was amplified with oligos oPAS204 and oPAS205. This is necessary as *Tet(M)* does not confer tetracycline resistance in *E. coli*. The promoter nifJp was amplified with oligos oPAS206 and oPAS207. Finally, *Tet(M)* was amplified with oligos oPAS208 and oPAS209. The four fragments were assembled into plasmid pVEL4 by Gibson Assembly.

### Promoter assessment

For CreiLOV promoter assessment, plasmid pVEL1 was digested with BsrGI to linearize and then assembled with the promoter and CreiLOV by Gibson Assembly to generate plasmids pEL2, pEL3, pEL4, and pEL5. Promoter nifJp was amplified from pVpas3 with oPAS210 and oPAS211 for pEL2. p2TetO1 and pThlA were synthesized with overhangs for pEL3 and pEL4, respectively, and J23119 was created via oligo annealing with oPAS216 and oPAS217 for pEL5. CreiLOV was used directly from synthesized DNA. Plasmids were confirmed with colony PCR and sequencing and transformed into *E. limosum*. Triplicate cultures were prepared according to Supplementary Table S3 and then sampled at 16 h, 24 h, and 40 h. Cultures were washed in PBS and 200 μl transferred to a 96 well plate and placed in a Molecular Devices SpectraMax i3 plate reader. Fluorescence was measured at an excitation of 450 nm and an emission of 500 nm. For each time point, we also collected and washed a second sample before aliquoting into 96 well plates and shaking vigorously for 6 h at 37°C to fully oxygenate. These samples were then measured in the same way as the anoxic ones.

For *catP* promoter assessment, plasmid pVEL2 was digested with BsrGI to linearize then assembled with the promoter and *catP* by Gibson Assembly to generate plasmids pEL2.1, pEL3.1, pEL4.1, and pEL5.1. Promoter nifJp was amplified from pVpas3 with oPAS210 and oPAS240 for pEL2.1, p2TetO1 was amplified from pEL3 with oPAS212 and oPAS241 for pEL3.1, pThlA was amplified from pEL4 with oPAS214 and oPAS242 for pEL4.1, and J23119 was created via oligo annealing with oPAS243 and oPAS255 for pEL5.1. *catP* was amplified from pVEL1 with oPAS238 and oPAS239. Plasmids were confirmed with colony PCR and sequencing and transformed into *E. limosum*. Triplicate cultures were prepared according to Supplementary Table S4 and allowed to grow to mid-exponential phase before patching 3 μl onto RCM agar plates containing thiamphenicol concentrations of 0, 25, 50, 100, 250, and 500 μg/ml. Plates were allowed to grow up for 3 days anoxically at 37°C before assessing growth. Next, each culture was collected by centrifugation at 20 000 g for 10 min at 4°C and washed in ice cold 50 mM Tris-HCl pH 7.4, pelleted again under the same conditions, and finally resuspended in 1 ml ice cold 50 mM Tris-HCl, pH 7.4, with 0.5 mg/ml lysozyme and HALT protease inhibitor cocktail obtained from Thermo Fisher Scientific. Samples were kept on ice and treated with beat beating. A total of 800 μl of cell suspension was transferred to a pre-prepared 2 ml bead tube obtained from Thomas Scientific, containing 0.1 mm glass acid washed beads. The tube was then placed on a Scientific Industries Vortex Genie 2 vortex mixer at max speed for 20 min to lyse cells. Lysate was centrifuged at 21 000 g for 15 min at 4°C and then used for the chloramphenicol acetyltransferase assay. Assay samples were prepared in biological triplicate in 96 well clear bottom assay plates. A total of 135 μl of 100 mM tris buffer pH 7.8, 5 μl of 2.5 mM 5,5ʹ-Dithiobis(2-Nitrobenzoic Acid) (DTNB), 5 μl of 5 mM acetyl Coenzyme A, and 2.5 μl of 3 mg/ml chloramphenicol were mixed together in each well and equilibrated at 25°C for 5 min until the absorbance at 412 nm stabilized. Then, 2.5 μl of each sample lysate was added and the change in A412 nm was observed for 3 min using kinetic measurement on a Molecular Devices SectraMax i3 plate reader. The maximum linear rate was calculated and used to determine promoter strength by normalizing against total protein concentration in the lysate as determined by the Pierce BCA protein assay.

## Results

### Implementation of new selectable markers

**Fig. 2.**
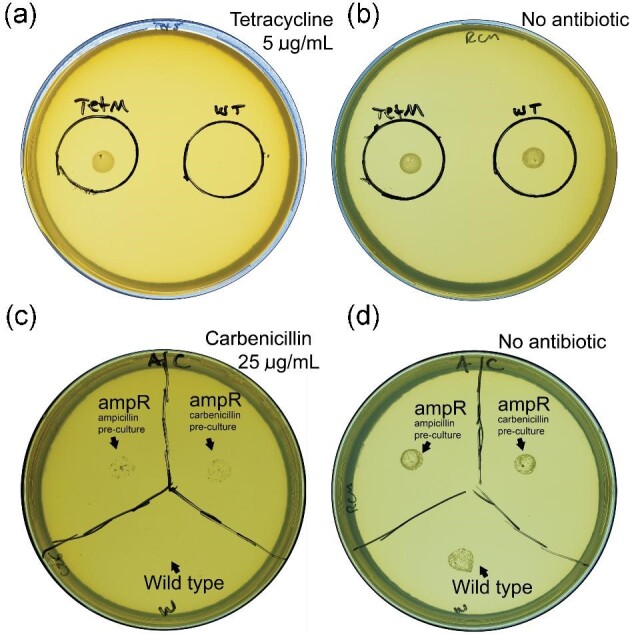
Selection marker assessment under optimal selective conditions. Antibiotic plates were prepared with the highest possible concentration of drug for which the engineered strain was resistant, and strains were grown up overnight in liquid culture before spotting 5 μl onto agar plates. (a) Growth of *tet(M)* strain on 5 μg/ml tetracycline while growth of wild type was totally inhibited (b) Both *tet(M)* and wild-type strains grow well on RCM plates without antibiotics. (c) Growth of *ampR* strain on 25 μg/ml carbenicillin while growth of wild type was totally inhibited. (d) Both *ampR* and wild-type strains grow well on RCM plates without antibiotics. On both plates, sections labeled ‘A’ were the *ampR* strain cultivated in liquid media with 5 μg/ml ampicillin, ‘C’ were cultivated in liquid media with 25 μg/ml carbenicillin prior to plating, and W is wild type. There appears to be no difference in resistance on solid media between strains propagated in ampicillin or carbenicillin.

*Eubacterium limosum* has two demonstrated selection makers: *ermB* for clarithromycin and *catP* for thiamphenicol. To develop additional resistance markers, we first examined susceptibility to additional antibiotics previously demonstrated to be effective against similar organisms by plating. We found that growth was fully inhibited by tetracycline, ampicillin, and carbenicillin (Lee et al., 2012; Watt, 1979). Then, we created a modified form of the pCL2 plasmid by replacing the small MCS and Lac regulatory elements with a significantly larger MCS more suited to enzymatic digestion, along with an rrnBt terminator. Next, we utilized this new plasmid architecture (pVEL1) to construct plasmids carrying antibiotic resistance genes for tetracycline and derivatives as well as ampicillin and derivatives under the control of the nifJp promoter, which is known to be very strong and constitutive, ensuring adequate expression of the resistance gene (Shin et al., 2019). Putative resistance genes were taken from sources as phylogenetically similar to *E. limosum* as possible to improve the likelihood of correct functionality. The tetracycline genes *tet(M)* and *tet(W)* confer resistance via ribosomal modification and were obtained from *Clostridium difficile* and *Butyrivibrio fibrisolvens*, respectively, the *tet(L)* gene confers resistance via efflux and was obtained from *Enterococcus faecalis*, and finally, the *ampR* gene confers resistance via ribosomal modification and was obtained from *B. subtilis* plasmid pCT5-bac1.8 (Ammor et al., 2008; Frazzon et al., 2010; Sebaihia et al., 2006). Constructs were transformed into *E. limosum* and then cells were plated onto selective agar media across a broad range of antibiotic, concentrations starting from our previously determined minimal inhibitory concentration for the wild-type strain. Strains expressing *ampR* and *tet(M)* were found to be resistant to their respective drugs. Strains carrying the *tet(L)* and *tet(W)* genes were not found to be resistant at any tetracycline concentration; however, *tet(M)* conferred resistance up through 5 μg/ml tetracycline, while the wild-type strain was inhibited down to 0.5 μg/ml (Fig. 2a and b and Supplementary Fig. S1). The *ampR* gene confers resistance to both ampicillin and carbenicillin as these two compounds are homologues, and strains with *ampR* were resistant to carbenicillin at concentrations up to 25 μg/ml. However, growth of the wild-type strain was only totally inhibited at 15 μg/ml, making the effective range of carbenicillin selection very narrow (Fig. 2c and d). Interestingly, the *ampR* gene did not confer any resistance to ampicillin on solid media; however, did confer resistance up to 5 μg/ml in liquid media. This makes ampicillin ineffective for cloning workflows, and only carbenicillin should be used in conjunction with the *ampR* gene to isolate single colonies on plates.

### Determination of plasmid copy number

**Fig. 3.**
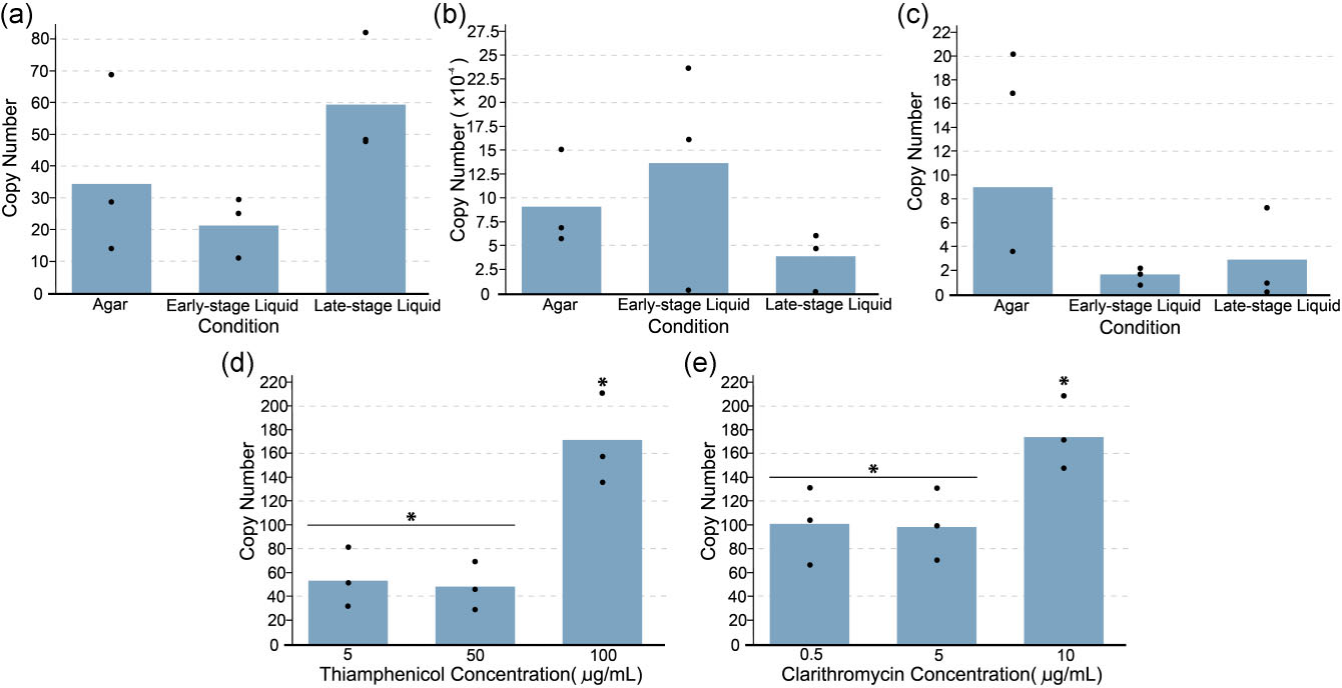
Plasmid copy number for different origins of replication across life cycle stages and antibiotic concentrations. Individual datapoints are shown in black: (a) pIP404 origin of replication from pCL2, (b) RepA origin of replication from pMTL82254, (c) RepL origin of replication from pMTL83151, (d) pIP404 copy number with thiamphenicol resistance (*catP* gene) in late-stage liquid culture with varying antibiotic concentrations. Significance was assessed using an unpaired *t*-test (*P < 0.05). (e) pIP404 copy number with clarithromycin resistance (*ermB* gene) in late-stage liquid culture with varying antibiotic concentrations. Significance was assessed using an unpaired *t*-test (*P < 0.05).

We used qPCR to quantify the copy number of plasmids harboring the pIP404, repA, and repL origins of replication when propagated in *E. limosum* at various stages in the bacterial life cycle. Our results support the finding that the pIP404 origin of replication is very active in *E. limosum*, with an average copy number of ∼60 copies per cell under normal liquid culture conditions (Fig. 3a). The origins repA and repL are less effective, with both possessing lower copy numbers over the lifecycle of a culture, with a maximum of ∼0.001 for RepA and 14 for RepL. Bizarrely, the copy number of pMTL82254 (repA) was significantly lower than 1 copy per cell, regardless of the condition or age of the culture.

Having ascertained that the pIP404 origin of replication has the highest copy number, we sought to test the plasmid's susceptibility to curing with both *catP* (thiamphenicol) and *ermB* (clarithromycin) selection markers. We grew cultures of *E limosum* carrying pCL2 (*catP*) and pCL2.1 (*ermB*) in Hungate tubes with 5 ml of DSMZ 135 media containing 50 μg/ml thiamphenicol and 5 μg/ml clarithromycin, respectively, until the early exponential phase. We then passaged a 1% inoculum from this liquid culture for each plasmid to a fresh tube with 5 ml of media and no antibiotic and cultured for 72 h to replicate the timescale of a typical bacterial fermentation for production of a heterologous product. We assessed plasmid curing by plating a 1:100 000 dilution of the culture onto plates containing the appropriate antibiotics and comparing against plates with no antibiotics. In both cases, we found that there was no loss of plasmid over this time course, which implies that the pIP404 origin of replication is highly stable (Table 1).

**Table 1.** Copy number for pIP404 origin of replication in pCL2 and pCL2.1 after 72 h growth without antibiotics. A *t*-test with a *P*-value of 0.05 found no significant difference between the CFU numbers on selective vs. unselective plates, indicating that the pIP404 origin of replication is extremely robust to curing

Plasmid	CFUs on RCM plate	CFUs on selective plate	Average Plasmid retention
pCL2	161	154	100.95%
pCL2	114	133	
pCL2	147	139	
pCL2.1	78	86	94.14%
pCL2.1	91	83	
pCL2.1	87	72	

Based on reports of the interplay between antibiotic concentration and plasmid copy number, we sought to determine if antibiotic selection marker and concentration have an impact on plasmid copy number for the pIP404 origin of replication in *E. limosum* (Millan et al., 2015). We grew triplicate cultures of *E. limosum* strains containing pCL2 and pCL2.1 plasmids with the pIP404 origin of replication to late-stage liquid culture with varying concentrations of thiamphenicol and clarithromycin, respectively. Cells were collected, lysed, and used for qPCR determination of plasmid copy number in the same way as for the initial copy number assessment. The copy number of pCL2 averaged ∼50 for thiamphenicol concentrations of 5 and 50 μg/ml and 170 for 100 μg/ml, whereas the copy number for pCL2.1 averaged ∼100 for 0.5 and 5 μg/ml clarithromycin but rose to ∼170 for 10 μg/ml. These findings indicate that reducing antibiotic concentration in liquid media below the normal selective concentration does not negatively impact copy number. However, in both cases, increasing the antibiotic concentration above the typical selective concentration trended toward a higher copy number regardless of the mode of action of the resistance gene (Fig. 3d and e). This finding may enable a new method of tuning gene expression for plasmid borne expression systems in *E. limosum*.

### Determination of oxygen resistance

**Fig. 4.**
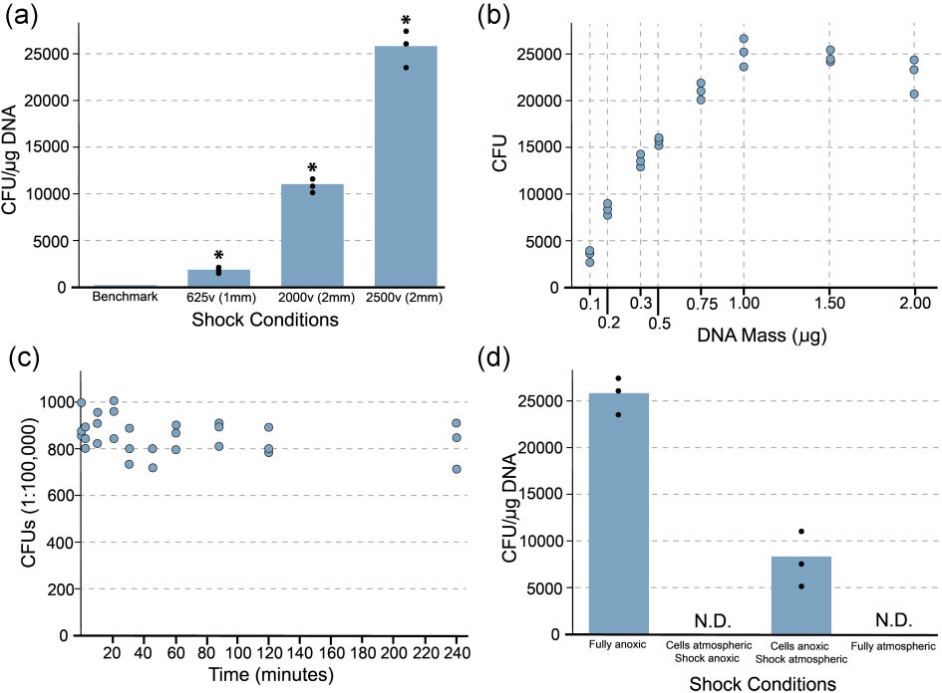
Development of atmospheric engineering techniques. (a) CFUs per μg of pCL2 plasmid DNA in 1 ml transformation reaction across different voltages and cuvette gap-widths. Transformations were done with 1 μg of DNA and run in triplicate in anoxic conditions. Individual datapoints are shown by black dots. Significance was assessed using an unpaired *t*-test (*P < 0.05). (b) Relationship between DNA mass and number of transformed cells using optimized protocol with 2500 v, 600 Ω, and 25 μF in a 2 mm cuvette in anoxic conditions. Transformations were performed in triplicate and all data is shown. (c) Scatterplot of the number of colonies counted on RCM plates from 1:100 000 dilutions of wild-type cultures exposed to atmospheric conditions for up to 4 h. All data are shown. Linear regression reveals an *R*^2^ value of 0.0171 for this dataset, indicating that there is no correlation between cell viability and exposure time to oxygen within the window we tested. (d) Transformation efficiency of *E. limosum* transformed with pCL2 plasmid under various oxygen exposure conditions. Transformations were done in triplicate and all datapoints are shown.

In order to ameliorate engineering difficulties in *E. limosum*, we sought to develop a transformation protocol that could be executed in aerobic conditions. We developed a simple and fast electrocompetency protocol that resulted in a maximum transformation efficiency of >2.5 × 10^4^ CFUs/μg of pCL2 plasmid DNA, more than two orders of magnitude more efficient than the previous best protocol. Competent cells are prepared by two centrifugation steps with washes in sucrose and sucrose/glycerol buffers and electroporation performed with an exponential decay protocol in a 2 mm gap-width cuvette at 2500 v, 600 Ω, and 25 μF (Fig. 4a).

We tested multiple different shock settings and found that a higher voltage with a larger gap-width cuvette resulted in higher transformation efficiencies. However, the competent cells were still transformed by most combinations of voltage and cuvette size. Additionally, we tested our preferred shock conditions across a panel of plasmid DNA concentrations from 0.1 μg to 2 μg of pCL2 plasmid DNA and found that optimal efficiency was obtained around 400 ng of DNA (final concentration ∼7.5 ng/μl) although overall number of transformants scaled mostly linearly with the amount of DNA used up to ∼1 μg where it saturated (Fig. 4b). Electrocompetent cells prepared in this way are viable for at least a month with no loss of efficiency, are agnostic to DNA concentration in the transformation mixture, and result in high-yield transformations across multiple different shock settings.

Next, we sought to adapt our protocol to atmospheric conditions. In all cases, fully anoxic media was required to sustain growth. However, when dilute cultures were exposed to atmospheric conditions for up to 4 h, they were able to survive (but not grow) with little to no loss of viability if returned to anoxic conditions and plated out on anoxic agar plates afterward (Fig. 4c). Based on this finding, we tested our electroporation protocol both in anoxic and atmospheric conditions with competent cells prepared both anoxically and atmospherically. We found that the shock step of transformation by electroporation could be executed outside the anaerobic chamber with ∼30% the efficiency of a fully anoxic process so long as the competent cells were prepared anoxically and transformed cells were immediately returned to an anoxic environment and recovered with anoxic media (Fig. 4d). However, all combinations with competent cells prepared atmospherically did not result in any transformants, indicating that there is a clear limit to the organism's oxygen tolerance. These results should bolster engineering efforts where some exposure to oxygen is unavoidable, such as during fluorescent assisted cell sorting (FACS) processes or in situations where specific pieces of equipment such as electroporators may not fit inside an anaerobic chamber.

### Development of a color-based genetic reporter assay

**Fig. 5.**
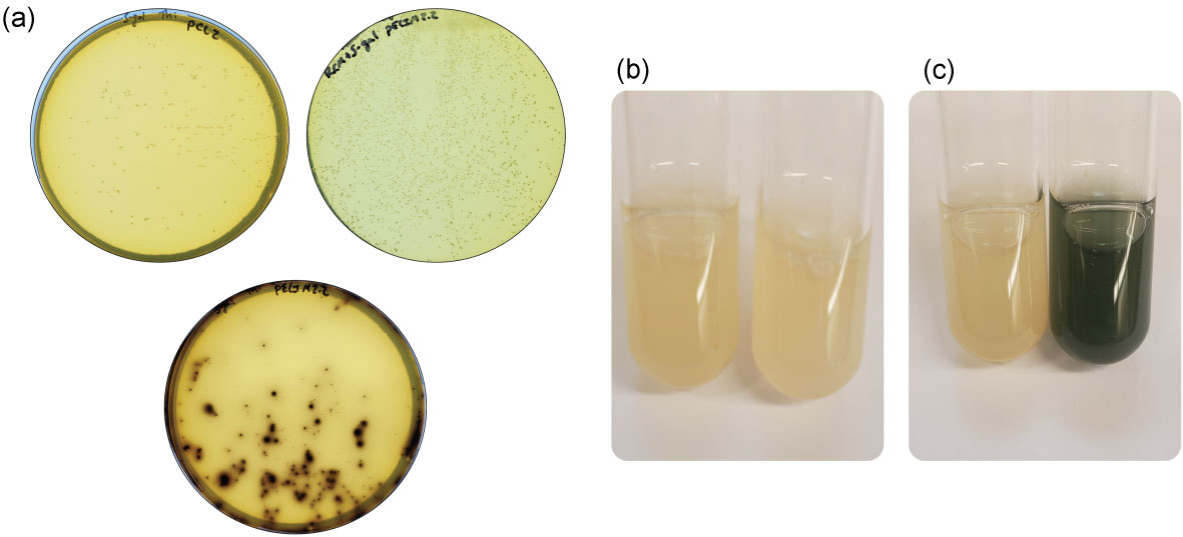
*BgaB* colorimetric reporter assay: (a) RCM agar plates after 3 days of incubation at 37°C. Top left: pCL2 strain on RCM + thiamphenicol agar plate with S-Gal and ammonium ferric sulfate. Top right: pELIM2.2 strain on RCM + thiamphenicol agar plate without S-Gal. Bottom: pELIM2.2 strain on RCM + thiamphenicol agar with S-Gal and ammonium ferric sulfate. Note how the colonies on only the bottom plate have turned black. (b) Liquid cultures of pCL2 carrying *E. limosum* (left) and *BgaB* expressing *E. limosum* (right) before induction with S-Gal and ammonium ferric sulfate. (c) Liquid cultures of pCL2 carrying *E. limosum* (left) and *BgaB* expressing *E. limosum* (right) 15 min after induction with S-Gal and ammonium ferric sulfate. Note how the *BgaB* tube is now black.

The traditional beta galactosidase blue/white screen is an excellent qualitative readout of gene expression; however, it is not applicable to *E. limosum* as it requires an aerobic environment (Juers et al., 2012). Recently, such a system was implemented in the soil dwelling, gram positive, thermophile *Geobacillus stearothermophilus* and shown to be functional under anaerobic conditions (Jensen et al., 2017). In this system, the beta galactosidase enzyme encoded by *BgaB* cleaves the lactose homologue S-Gal, the product of which oxidizes in the presence of ammonium ferric sulfate to create an insoluble black product. We imported this system into *E. limosum* by codon harmonizing the gene and cloning it into the pCL2 backbone under the control of the nifJp promoter to make the *BgaB* expression plasmid pELIM2.2. Then, *E. limosum* strains harboring the plasmid were plated onto RCM agar plates containing S-Gal and ammonium ferric sulfate alongside the strain harboring the empty vector. After 3 days of incubation at 37°C the colonies carrying *BgaB* turned black on the plate containing S-Gal, whereas the empty vector colonies did not change color on the S-Gal plate and neither did the *BgaB* expressing strain on a plate without S-Gal (Fig. 5a). We also tested whether the assay could be used in liquid cultures. We grew up identical cultures of empty vector and *BgaB* carrying strains overnight, then added identical aliquots of S-Gal and ammonium ferric sulfate. After 15 min incubating at 37°C, the *BgaB* tube changed color to black, and there was no color change observed in the empty vector tube (Fig. 5b and c). This rapid and versatile system will enable quick and easy gene expression screening in *E. limosum*.

### Promoter assessment

Several promoter systems have been reported in *E. limosum*; however, specifics of their strength and dynamic range have not been reported (Flaiz et al., 2021; Shin et al., 2019). Here, we selected a panel of promoters previously described to be functional in *E. limosum* and quantitatively assessed their strength. We initially attempted to screen promoters using a fluorescent protein strategy utilizing the anaerobic CreiLOV reporter protein but found that it was not suitable for a quantitative assessment of promoter function (Mukherjee et al., 2015). Specifically, we observed very poor fluorescence range when compared to autofluorescence, a fluorescence signal that reduced over time even though the culture remained healthy, and further, these results were not improved by oxygen rescue to aid protein maturation (Supplementary Fig. S2). Based on these findings, we decided to pursue a different quantification strategy for promoter strength. The *catP* gene encodes the enzyme chloramphenicol acetyltransferase, which acts directly on the antibiotic drug and therefore can be used as a proxy for gene expression with strains expressing higher levels of *catP* capable of surviving on higher concentrations of chloramphenicol or thiamphenicol (Heap et al., 2009). Expression of *catP* can also be quantified by an absorbance assay in parallel relying on the acetylation of chloramphenicol by the activity of the enzyme, with more acetylation correlating to higher gene expression (Davis and White, 2002). We placed *catP* under the control of four different promoters: constitutive promoters nifJp, pThlA, and J23119; and inducible promoter p2TetO1 controlled by anhydrotetracycline (aTc) within the pVEL1 plasmid architecture, utilizing *ermB* as the selection marker. We transformed these into *E. limosum* and measured the gene expression by these two metrics.

**Fig. 6.**
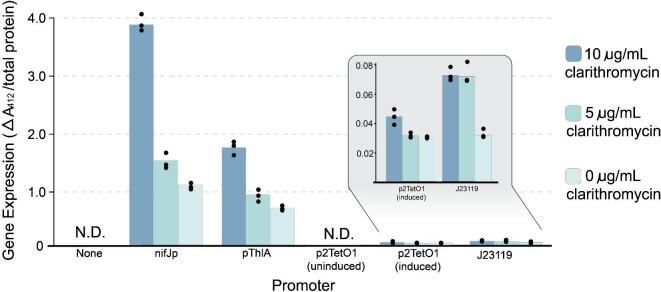
Gene expression as measured by the chloramphenicol acetyltransferase enzymatic assay. A greater change in absorbance at 412 nm corresponds to more chloramphenicol acetyltransferase enzyme present, and therefore greater promoter strength or gene expression. Strains were maintained at three different antibiotic concentrations to simultaneously assess the impact of plasmid copy number on gene expression. Conditions at 0 μg/ml clarithromycin are representative of general ‘promoter strength’ and the conditions at 5 μg/ml and 10 μg/ml clarithromycin demonstrate how gene expression can be increased by increasing plasmid copy number by adding clarithromycin. These data indicate that gene expression increases as copy number increases, meaning that plasmid-borne expression is tunable by clarithromycin concentration. Further, these results indicate that this trend is mostly agnostic to the promoter driving the gene of interest, and therefore the change in gene expression level is a function only of the change in plasmid copy number.

First, we grew up overnight cultures of the promoter strains, both with and without aTc, and patched 3 μl onto plates with increasing thiamphenicol concentrations of 0, 25, 50, 100, 250, and 500 μg/ml. The growth patterns on the plates indicate that promoters nifJp and pThlA are constitutive and very strong, J23119 is weak and constitutive, and p2TetO1 is inducible by aTc with very tight regulation but its strength is slightly weaker than even J23119 (Supplementary Fig. S3). For a quantitative assessment, clarified lysates from liquid cultures of each of the promoter strains were prepared and used for the spectrophotometric determination of chloramphenicol acetyltransferase activity (Shaw, 1975). The cultures for this assay were prepared without antibiotics to get an unbiased readout for promoter strength in the absence of any selective pressure, as this is likely how the promoters will be used in large-scale fermentation. From this assay, we observed an almost identical pattern as in the patching assay (Fig. 6). Additionally, this assay provides more granularity and reveals that pThlA is about half as strong as nifJp, with this difference being lost somewhere between 250 μg/ml and 500 μg/ml thiamphenicol in the patching assay. The regulation of p2TetO1 is tight, with biological triplicate measurements detecting no acetyl-CoA utilization during the assay for uninduced conditions. The induced p2TetO1 promoter and the J23119 promoter have similar strengths, both with expression ∼36-fold lower than nifJp.

To further explore the finding of antibiotic concentration affecting plasmid copy number—and therefore gene expression—we expanded the promoter screening to include cultures maintained at different antibiotic concentrations. We grew up cultures of strains carrying our promoter assessment plasmids on both 5 μg/ml and 10 μg/ml clarithromycin and then performed the chloramphenicol acetyltransferase spectrophotometric assay to evaluate gene expression. We found that in almost all cases, the amount of protein product increased with increasing clarithromycin concentration, demonstrating the ability to dynamically tune gene expression via copy number modulation induced by changes in antibiotic concentration (Fig. 6). We also noticed that despite the theoretical increase in metabolic burden at higher antibiotic concentrations and higher plasmid copy numbers, the cultures at the highest antibiotic concentration reached the same endpoint OD_600_ as the cultures with lower concentrations. These results indicate that varying antibiotic concentration can be used as an additional method to fine-tune expression in *E. limosum.* Overall, this detailed promoter assessment will enable researchers to make effective promoter choices for future engineering efforts.

## Discussion

We were able to develop many new beneficial engineering tools for *E. limosum*, which should significantly benefit its tractability for engineering. In regard to the plasmid copy number, we selected our panel to screen with qPCR based on the several plasmids that have been used for engineering *E. limosum* in the past (Flaiz et al., 2021; Heap et al., 2009; Leang et al., 2013; Shin et al., 2019). Some initial work has been done to assess the relative strength of origins of replication across plasmids of interest. However, this was measured by the transformation efficiency of the relevant plasmids and is not sufficient for a full understanding of the copy number of each origin of replication. Our results showing that the pIP404 origin of replication has the highest copy number is in line with these previously published qualitative results. During our analysis, we also observed that the copy number for pMTL82254 was much below one in all cases. The pMTL82254 plasmid encodes the *ermB* gene for resistance against clarithromycin and the repA origin of replication. The *ermB* resistance acts by methylating the ribosome to prevent the antibiotic acting upon it. We hypothesize that cells may be cheating selection via methylated ribosomes passed to daughter cells during division, potentially conferring resistance to many cells which have been cured of the plasmid (Leclercq, 2002). This may possibly explain why so many cells in the population lacked the necessary plasmid but still were capable of evading clarithromycin toxicity. This hypothesis is, at this point, little more than conjecture and must be tested to understand the mechanism of such a low copy number. However, previous qualitative evaluations of this origin of replication have found it to be almost non-functional in *E. limosum* (Jin et al., 2020; Shin et al., 2019). Therefore, it is probable that we succeeded in propagating strains carrying this plasmid simply because we utilized a transformation protocol many orders of magnitude more efficient than previous studies and thus were able to obtain a suitable number of transformants for experimentation.

In regard to this transformation protocol, low transformation efficiencies have long been a bottleneck in engineering acetogens, and therefore it is unsurprising that we obtained these results regarding the repA origin of replication (Bourgade et al., 2021). *Eubacterium limosum* not only suffers from low transformation efficiency, but it has also been largely neglected in terms of optimizing transformation protocols. Most recent reports of engineering in *E. limosum* have used modified protocols originally derived from a method for *Clostridium spp.* (Flaiz et al., 2021; Leang et al., 2013; Shin et al., 2019). These protocols are slow and cumbersome due to the complex buffers involved, are more prone to arcing due to the presence of salts and have low overall efficiency with a maximum reported efficiency of 1.4 × 10^2^ transformants per μg of plasmid DNA. The protocol reported here has an efficiency more than two orders of magnitude greater, and therefore enables techniques, which were previously not possible, such as using the repA origin of replication. During preparation of this manuscript, a similar electrocompetency protocol with comparable efficiency was published (Song et al., 2022). Our protocol varies slightly from theirs by differences in centrifugation speed and time and requires one fewer wash step.

To evaluate new antibiotic selection markers, we leveraged *E. limosum*’s ability to grow well on agar plates—a trait not shared by most other acetogens, including the model acetogen *Clostridium ljungdhalii* (Molitor et al., 2016). This propensity to plate well was invaluable in many aspects of tool development, and most particularly in selection marker development. During selection marker screening, we observed several differences between ampicillin and carbenicillin. The *ampR* gene was incapable of conferring resistance to ampicillin on solid media, but was very effective in liquid culture, whereas carbenicillin required higher concentrations for selection but was effective in both solid and liquid media. Both antibiotics are beta-lactams with a nearly identical structure and the same mode of action. The differences in selective concentration between the two are unsurprising given similar findings in other bacteria (Russell and Mills, 1974). The differences between ampicillin on solid and liquid media, however, seem to indicate that the biology of *E. limosum* undergoes a significant change depending on physical conditions. Other related anaerobic bacteria have been observed to possess some native resistance capabilities against beta-lactams, which seem to be regulated by their life cycle and environment (Nord, 1986). While we cannot hypothesize on what the extent of these changes might be in *E. limosum*, these observations warrant additional investigation to further understand the fundamental biology of the organism. In the short term, these observations should provide caution to researchers seeking to use beta lactams with *E. limosum*.

During our initial attempts to evaluate promoters in *E. limosum* using the fluorescent CreiLOV protein, we observed evidence that certain *E. limosum* promoters work in *E. coli* by observing color change and fluorescence signal from within the *E. coli* cloning vector (Supplementary Fig. S4). Upon performing DNA sequencing on colonies of different colors, we noticed that the strongest promoters were very likely to become mutated in such a way where protein expression was disrupted. These results may explain some of the cloning difficulties we faced during other tool development work, as relatively high expression of *E. limosum* genes in *E. coli* may be toxic and therefore select for mutated constructs during cloning. Development of orthogonal promoters with no activity in *E. coli* could facilitate further genetic engineering work.

During the promoter assessment, we used two different strategies: patching and enzymatic assay. Antibiotic concentration screening is a traditional method for assessing gene expression in many organisms, especially by agar plate patching assays. By performing a plating assay alongside the spectrophotometric assay, we validated the patching approach with the *catP* gene in *E. limosum*. In situations where a qualitative readout is sufficient, performing a plating assay may be more expedient than protein extraction and spectrophotometric analysis. Our analysis of the *catP* gene for this application also opens the door for its use in more advanced engineering strategies such as directed evolution, where the gene may be tied to cell viability and used as a leaver for controlling cell populations with advantageous mutations tied to *catP* expression.

By developing these new tools, we have expanded the foundational engineering apparatus for *E. limosum* and broadened the scope and speed at which research can be conducted on this organism. In summary, we implemented a colorimetric genetic screening assay, which is the first time this has been accomplished in an acetogen. Further, we assessed oxygen tolerance for bench work outside of anoxic conditions and for the first time determined the copy number of common acetogenic plasmid origins of replication. We quantitatively assessed the strength of a panel of promoters and report the discovery of two new antibiotic selection markers that were previously not known to function in *E. limosum—*doubling the number of antibiotic selection systems currently available for this organism. These advances should improve the tractability of this microbe and further simplify its use for metabolic engineering and synthetic biology.

## Supplementary Material

kuac019_Supplemental_File

## References

[bib1] Ammor M.S. , Flórez A.B., Álvarez-Martín P. et al. (2008). Analysis of tetracycline resistance tet (W) genes and their flanking sequences in intestinal Bifidobacterium species. Journal of Antimicrobial Chemotherapy 62(4), 688–693. 10.1093/jac/dkn280. 18614524

[bib2] Bourgade B. , Minton N.P., Islam M.A. (2021). Genetic and metabolic engineering challenges of C1-gas fermenting acetogenic chassis organisms. FEMS Microbiology Reviews 45(2), 1–20. 10.1093/femsre/fuab008. PMC8351756 33595667

[bib3] Claassens N.J. , Siliakus M.F., Spaans S.K. et al. (2017). Improving heterologous membrane protein production in Escherichia coli by combining transcriptional tuning and codon usage algorithms. PLoS ONE 12(9), e0184355. https://doi.org/576 10.1371/journal.pone.0184355. 28902855 10.1371/journal.pone.0184355 PMC5597330

[bib4] Cotton C.A. , Claassens N.J., Benito-Vaquerizo S. et al. (2020). Renewable methanol and formate as microbial feedstocks. Current Opinion in Biotechnology 62, 168–180. 10.1016/j.copbio.2019.10.002. 31733545

[bib5] Davis J.R.E. , White M.R.H. (2002). Chloramphenicol acetyl transferase. In: Jacqueline I. Kroschwitz (ed.), Wiley Encyclopedia of Molecular Medicine. New York, NY: Wiley. 10.1002/0471203076.emm1201.

[bib6] Drake H.L. , Daniel S.L., Küsel K. et al. (1997). Acetogenic bacteria: what are the in situ consequences of their diverse metabolic versatilities. BioFactors 6(1), 13–24. 10.1002/biof.5520060103. 9233536

[bib7] Flaiz M. , Ludwig G., Bengelsdorf F.R. et al. (2021). Production of the biocommodities butanol and acetone from methanol with fluorescent FAST-tagged proteins using metabolically engineered strains of Eubacterium limosum. Biotechnology for Biofuels 14(1), 1–20. 10.1186/s13068-021-01966-2. 33971948 PMC8111989

[bib8] Frazzon A.P.G. , Gama B.A., Hermes V. et al. (2010). Prevalence of antimicrobial resistance and molecular characterization of tetracycline resistance mediated by tet(M) and tet(L) genes in Enterococcus spp. isolated from food in Southern Brazil. World Journal of Microbiology and Biotechnology 26(2), 365–370. 10.1007/s11274-009-0160-x.

[bib9] Heap J.T. , Pennington O.J., Cartman S.T. et al. (2009). A modular system for Clostridium shuttle plasmids. Journal of Microbiological Methods 78(1), 79–85. 10.1016/j.mimet.2009.05.004. 19445976

[bib10] Jensen T.Ø. , Pogrebnyakov I., Falkenberg K. B. et al. (2017). Application of the thermostable β-galactosidase, BgaB, from Geobacillus stearothermophilus as a versatile reporter under anaerobic and aerobic conditions. AMB Express 7(1), 169. 10.1186/s13568-017-0469-z. 28875485 PMC5585113

[bib11] Jin S. , Bae J., Song Y. et al. (2020). Synthetic biology on acetogenic bacteria for highly efficient conversion of C1 gases to biochemicals. International Journal of Molecular Sciences 21(20), 1–25. 10.3390/ijms21207639. PMC7589590 33076477

[bib12] Juers D.H. , Matthews B.W., Huber R.E. (2012). LacZ β-galactosidase: structure and function of an enzyme of historical and molecular biological importance. Protein Science 21(12), 1792–1807. 10.1002/pro.2165. 23011886 PMC3575911

[bib13] Kelly W.J. , Henderson G., Pacheco D.M. et al. (2016). The complete genome sequence of Eubacterium limosum SA11, a metabolically versatile rumen acetogen. Standards in Genomic Sciences 11(1), 1–10. 10.1186/s40793-016-0147-9. 26981167 PMC4791908

[bib14] Kremp F. , Müller V. (2021). Methanol and methyl group conversion in acetogenic bacteria: biochemistry, physiology and application. FEMS Microbiology Reviews 45(2), 1–22. 10.1093/femsre/fuaa040. 32901799

[bib15] Leang C. , Ueki T., Nevin K.P. et al. (2013). A genetic system for Clostridium ljungdahlii: a chassis for autotrophic production of biocommodities and a model homoacetogen. Applied and Environmental Microbiology 79(4), 1102–1109. 10.1128/AEM.02891-12. 23204413 PMC3568603

[bib16] Leclercq R. (2002). Mechanisms of resistance to macrolides and lincosamides: nature of the resistance elements and their clinical implications. Clinical Infectious Diseases 34(4), 482–492. 10.1086/324626. 11797175

[bib17] Lee C. , Kim J., Shin S.G. et al. (2006). Absolute and relative QPCR quantification of plasmid copy number in Escherichia coli. Journal of Biotechnology 123(3), 273–280. 10.1016/j.jbiotec.2005.11.014. 16388869

[bib18] Lee M.R. , Huang Y.T., Liao C.H. et al. (2012). Clinical and microbiological characteristics of bacteremia caused by Eggerthella, Paraeggerthella, and Eubacterium species at a University Hospital in Taiwan from 2001 to 2010. Journal of Clinical Microbiology 50(6), 2053–2055. 10.1128/JCM.00548-12. 22495556 PMC3372111

[bib19] Loubiere P. , Gros E., Paquet V. et al. (1992). Kinetics and physiological implications of the growth behaviour of Eubacterium limosum on glucose/methanol mixtures. Journal of General Microbiology 138(5), 979–985. 10.1099/00221287-138-5-979.

[bib20] Millan A.S. , Santos-Lopez A., Ortega-Huedo R. et al. (2015). Small-plasmid-mediated antibiotic resistance is enhanced by increases in plasmid copy number and bacterial fitness. Antimicrobial Agents and Chemotherapy 59(6), 3335–3341. 10.1128/AAC.00235-15. 25824216 PMC4432117

[bib21] Molitor B. , Kirchner K., Henrich A.W. et al. (2016). Expanding the molecular toolkit for the homoacetogen Clostridium ljungdahlii. Science Reports 6, 1–10. 10.1038/srep31518. PMC4985741 27527841

[bib22] Mukherjee A. , Lordan C., Ross R.P. et al. (2020). Gut microbes from the phylogenetically diverse genus Eubacterium and their various contributions to gut health. Gut Microbes 12(1), 1802866. 10.1080/19490976.2020.1802866. 32835590 PMC7524325

[bib23] Mukherjee A. , Weyant K.B., Agrawal U. et al. (2015). Engineering and characterization of new LOV-based fluorescent proteins from chlamydomonas reinhardtii and vaucheria frigida. ACS Synthetic Biology 4(4), 371–377. 10.1021/sb500237x. 25881501

[bib24] Müller V. (2019). New horizons in cetogenic conversion of one-carbon substrates and biological hydrogen storage. Trends in Biotechnology 37(12), 1344–1354. 10.1016/j.tibtech.2019.05.008. 31257058

[bib25] Nord C.E. (1986). Mechanisms of B-lactam resistance in anaerobic bacteria. Reviews of Infectious Diseases 8(S5), S503–S511. 3541135 10.1093/clinids/8.supplement_5.s543

[bib26] Roh H. , Ko H.J., Kim D. et al. (2011). Complete genome sequence of a carbon monoxide-utilizing acetogen, Eubacterium limosum KIST612. Journal of Bacteriology 193(1), 307–308. 10.1128/JB.01217-10. 21036996 PMC3019962

[bib27] Russell A.D. , Mills A.P. (1974). Comparative sensitivity and resistance of some strains of Pseudomonas aeruginosa and Pseudomonas stutzeri to antibacterial agents. Journal of Clinical Pathology, 27(6), 463–466. 10.1136/jcp.27.6.463. 4369876 PMC478156

[bib28] Sanford P.A. , Woolston B.M. (2022). Synthetic or natural? Metabolic engineering for assimilation and valorization of methanol. Current Opinion in Biotechnology 74, 171–179. 10.1016/j.copbio.2021.12.001. 34952430

[bib29] Schrader J. , Schilling M., Holtmann D. et al. (2009). Methanol-based industrial biotechnology: current status and future perspectives of methylotrophic bacteria. Trends in Biotechnology 27(2), 107–115. 10.1016/j.tibtech.2008.10.009. 19111927

[bib30] Schuchmann K. , Müller V. (2014). Autotrophy at the thermodynamic limit of life: a model for energy conservation in acetogenic bacteria. Nature Reviews Microbiology 12(12), 809–821. https://669 doi.org/10.1038/nrmicro3365. 25383604 10.1038/nrmicro3365

[bib31] Sebaihia M. , Wren B.W., Mullany P. et al. (2006). The multidrug-resistant human pathogen Clostridium difficile has a highly mobile, mosaic genome. Nature Genetics 38(7), 779–786. 10.1038/ng1830. 16804543

[bib32] Shaw W.V. (1975). Chloramphenicol acetyltransferase from chloramphenicol-resistant bacteria. Methods in Enzymology 43(C), 737–755. 10.1016/0076-6879(75)43141-X. 1094240

[bib33] Shin J. , Kang S., Song Y. et al. (2019). Genome engineering of eubacterium limosum using expanded genetic tools and the CRISPR-Cas9 system. ACS Synthetic Biology 8(9), 2059–2068. 10.1021/acssynbio.9b00150. 31373788

[bib34] Song Y. , Bae J., Jin S. et al. (2022). Development of highly characterized genetic bioparts for efficient gene expression in CO 2-fixing Eubacterium limosum. Metabolic Engineering 72(March), 215–226 10.1016/j.ymben.2022.03.016. 35364280

[bib35] Venkata Mohan S. , Modestra J.A., Amulya K. et al. (2016). A circular bioeconomy with biobased products from CO2 sequestration. Trends in Biotechnology 34(6), 506–519. 10.1016/j.tibtech.2016.02.012. 27048926

[bib36] Watt B. (1979). Antibiotic susceptibility of anaerobic bacteria. Journal of Infection 1(SUPPL. 1), 39–48. 10.1016/S0163-4453(79)80042-0.

[bib37] Yishai O. , Lindner S.N., Gonzalez de la Cruz J. et al. (2016). The formate bio-economy. Current Opinion in Chemical Biology 35, 1–9. 10.1016/j.cbpa.2016.07.005. 27459678

