# Supplementary material for: Expanding the genetic engineering toolbox for the metabolically flexible acetogen Eubacterium limosum
Source: J Ind Microbiol Biotechnol. 2022 Jul 26;49(5):kuac019. doi: 10.1093/jimb/kuac019 (PMC9559302; doi:10.1093/jimb/kuac019)
Supplement: kuac019_Supplemental_File [file kuac019_supplemental_file.docx]

**Expanding the genetic engineering toolbox for the metabolically flexible acetogen *Eubacterium limosum* – supplementary information**

Patrick A. Sanford and Benjamin M. Woolston


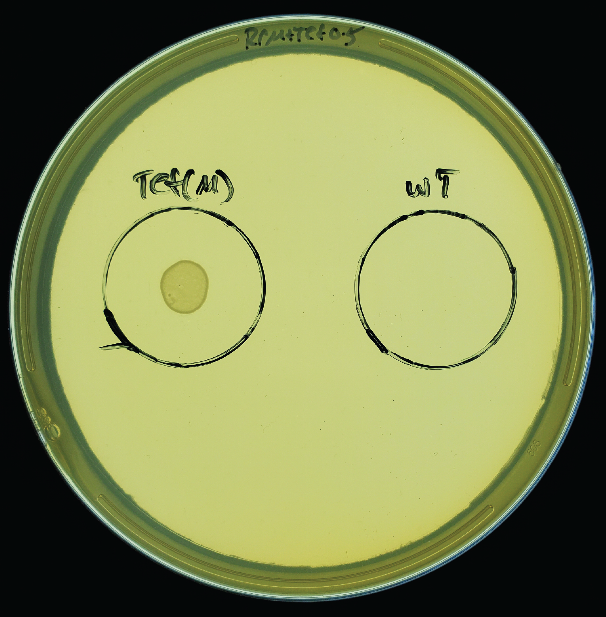


**Figure S1:** *E. limosum*, both wild type (WT) and carrying the tetracycline resistance gene *Tet(M)*, plated onto 0.5 μg/mL tetracycine plates. Note how even at this low concentration the growth of the wild type strain is totally inhibited.

**
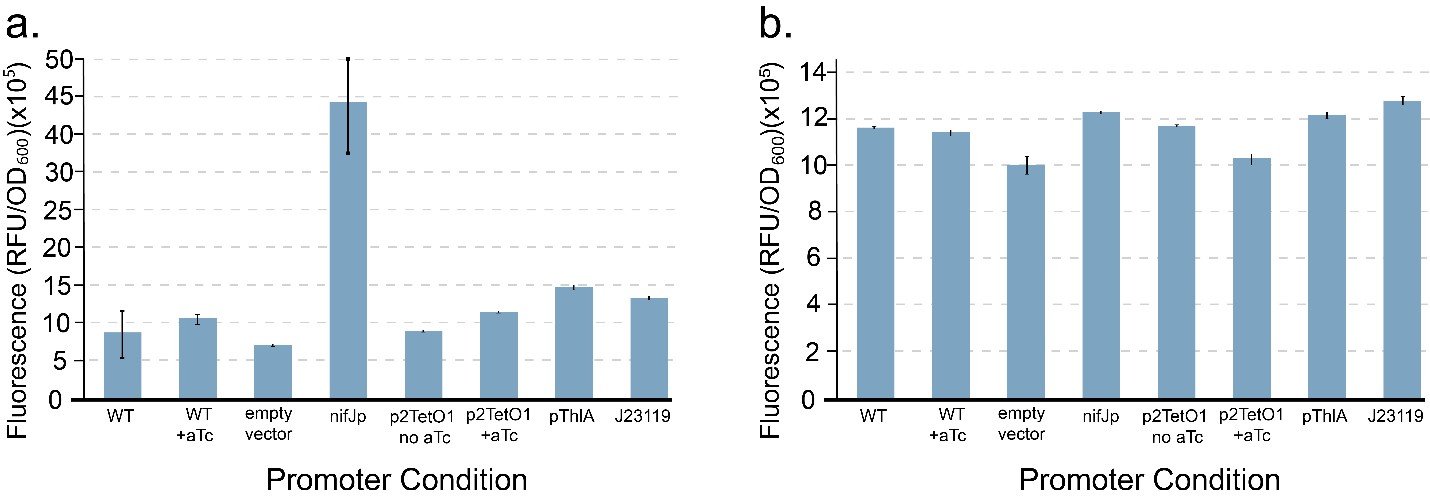
**

**Figure S2:** *E. limosum* CreiLOV fluorescence. **a)** Normalized fluorescence after 16 hours. Note the poor dynamic range of all the promoters in regard to the wild type strain. The activities of p2TetO1, pThlA, and J23119 are nearly undetectable. **b)** Normalized fluorescence after 40 hours. Note how at this point all strains have nearly identical fluorescence readouts to the wild type strain indicating an absence of the CreiLOV protein. Error bars indicate the standard deviation across biological triplicate cultures.

**
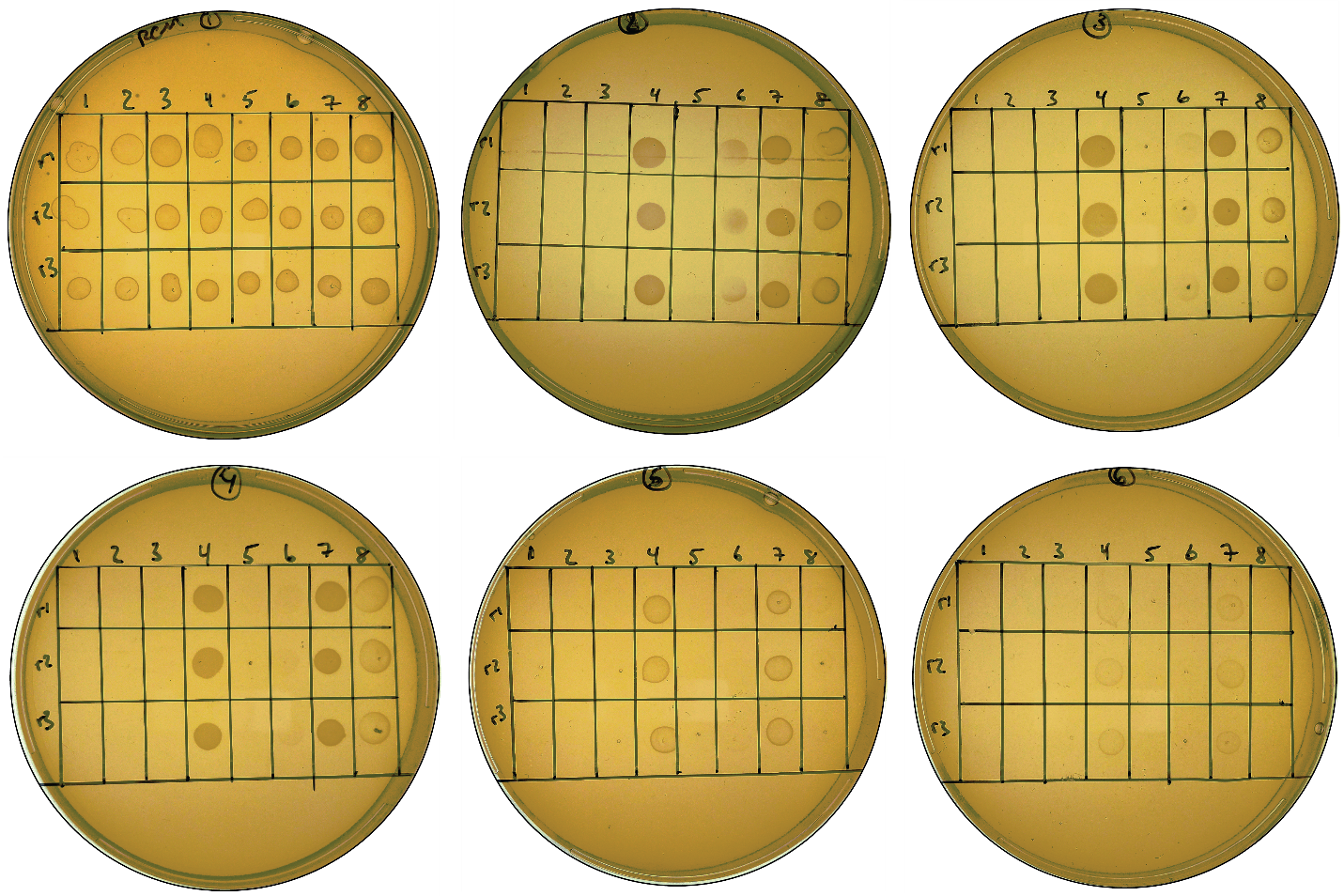
**

**Figure S3:** Promoter strengths as measured by thiamphenicol titration plating with strains plated on increasing concentrations of thiamphenicol. More strongly expressed catP genes will result in higher tolerance to thiamphenicol and therefore growth on higher concentration plates. Plates are in order of increasing concentration left to right top to bottom with 0, 25, 50, 100, 250, and 500 μg/mL respectively. 1=WT, 2=WT induced with aTc, 3=empty vector, 4=nifJp, 5=p2TetO1 uninduced, 6=p2TetO1 induced with aTc, 7=pThlA, 8=J23119. Each plate was patched from biological triplicate liquid cultures.

**
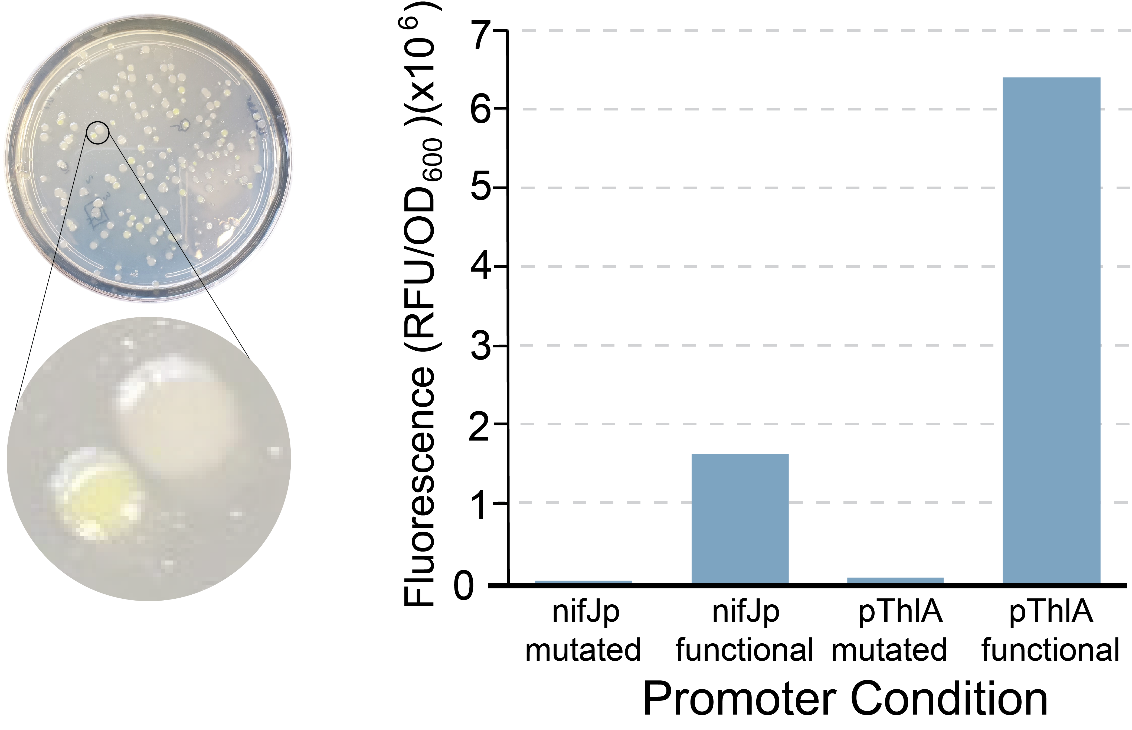
**

**Figure S4:** *E. coli* CreiLOV fluorescence. During the cloning for the CreiLOV *E. limosum* expression constructs we noticed that some of the colonies of the *E. coli* cloning host were changing color to green, but that others were white (left). Upon DNA sequencing we noticed that the white colonies had mutations in the promoters and that the green colonies were unmutated. The mutated colonies did not have high fluorescence, but the functional ones did, indicating that the nifJp and pThlA promoters are functional in *E. coli*.

**BgaB gene sequence:**

ATGAACGTTCTGTCCTCAATCTGCTACGGAGGAGATTACAACCCTGAGCAATGGCCAGAGGAAATTTGGTATGAAGACGCTAAGTTGATGCAAAAAGCGGGGGTGAATTTAGTGTCTTTAGGGATTTTCAGTTGGAGCAAAATCGAACCGTCTGATGGAGTGTTCGACTTTGAATGGCTCGACAAGGTTATAGACATACTATATGACCACGGTGTGTATATCAACTTGGGGACGGCGACCGCAACCACTCCGGCTTGGTTTGTAAAAAAATATCCGGATTCTTTGCCGATCGATGAAAGCGGAGTCATTCTCTCGTTTGGCAGCCGCCAACATTATTGTCCTAATCATCCTCAATTAATTACGCACATAAAGAGACTTGTGAGGGCTATTGCCGAACGGTATAAAAATCATCCGGCACTCAAAATGTGGCATGTTAACAATGAGTATGCATGTCACGTTTCCAAATGTTTTTGCGAGAATTGCGCCGTCGCGTTTCGGAAGTGGCTAAAGGAAAGATATAAAACAATCGACGAACTTAATGAACGTTGGGGTACAAACTTTTGGGGACAGCGATACAACCATTGGGATGAAATCAATCCCCCTAGAAAGGCACCGACGTTTATCAATCCAAGCCAGGAACTTGACTACTACCGTTTTATGAATGACTCAATTCTCAAGTTGTTTTTAACAGAAAAAGAAATTTTACGCGAGGTAACACCAGATATTCCGGTGTCAACTAATTTCATGGGTTCATTCAAACCGCTGAACTATTTTCAATGGGCGCAGCATGTAGATATTGTGACATGGGACTCATATCCTGACCCCAGAGAGGGCCTTCCGATCCAGCACGCCATGATGAATGACCTTATGCGTTCTCTTAGAAAAGGCCAACCGTTTATTTTGATGGAGCAGGTAACCTCGCATGTTAACTGGCGCGATATTAATGTTCCAAAACCGCCAGGTGTAATGCGTTTATGGAGTTATGCAACGATTGCCCGTGGCGCCGATGGTATTATGTTTTTCCAGTGGCGGCAAAGTAGAGCAGGAGCTGAAAAATTCCACGGTGCAATGGTGCCTCACTTTTTGAACGAGAATAATAGAATTTATCGCGAAGTTACACAGCTTGGACAAGAGCTGAAAAAGCTGGATTGTTTGGTCGGATCTAGAATCAAGGCCGAGGTCGCGATCATCTTTGATTGGGAAAACTGGTGGGCTGTCGAACTGTCCTCCAAACCGCATAACAAACTGCGCTATATTCCTATAGTTGAAGCTTATTACAGGGAATTATATAAACGGAATATTGCTGTCGATTTTGTCCGCCCATCTGATGATCTAACAAAATACAAAGTGGTTATCGCCCCAATGTTATATATGGTCAAAGAGGGAGAAGATGAAAACCTTCGGCAATTTGTTGCGAACGGCGGCACTCTGATTGTCAGCTTCTTCTCGGTCATCGTCGACGAAAATGACCGAGTACATCTCGGCGGATATCCTGGCCCTCTGCGAGATATTTTGGGCATCTTTGTTGAGGAATTTGTACCTTACCCGGAAACCAAAGTAAACAAAATCTATAGCAACGATGGCGAATATGATTGCACGACGTGGGCGGACATAATCCGGTTAGAAGGGGCAGAACCTCTCGCGACATTTAAGGGGGATTGGTATGCAGGACTTCCGGCGGTTACACGTAACTGCTACGGTAAAGGAGAGGGGATCTACGTCGGTACGTATCCGGATAGCAATTATTTAGGCAGGCTTTTAGAACAGGTCTTCGCTAAACATCATATTAACCCCATTCTTGAAGTAGCTGAAAATGTAGAGGTGCAGCAAAGAGAGACTGATGAATGGAAGTATCTTATTATTATCAATCATAATGATTACGAAGTGACACTGTCACTGCCGGAAGATAAAATATACCAGAATATGATTGATGGGAAATGTTTTCGAGGAGGCGAACTGAGGATTCAAGGCGTTGATGTGGCAGTGCTGCGCGAGCATGATGAAGCCGGCAAAGTTTAA

**AmpR gene sequence:**

ATGAGTATTCAACATTTCCGTGTCGCCCTTATTCCCTTTTTTGCGGCATTTTGCCTTCCTGTTTTTGCTCACCCAGAAACGCTGGTGAAAGTAAAAGATGCTGAAGATCAGTTGGGTGCACGAGTGGGTTACATCGAACTGGATCTCAACAGCGGTAAGATCCTTGAGAGTTTTCGCCCCGAAGAACGTTTTCCAATGATGAGCACTTTTAAAGTTCTGCTATGTGGCGCGGTATTATCCCGTGTTGACGCCGGGCAAGAGCAACTCGGTCGCCGCATACACTATTCTCAGAATGACTTGGTTGAGTACTCACCAGTCACAGAAAAGCATCTTACGGATGGCATGACAGTAAGAGAATTATGCAGTGCTGCCATAACCATGAGTGATAACACTGCGGCCAACTTACTTCTGACAACGATCGGAGGACCGAAGGAGCTAACCGCTTTTTTGCACAACATGGGGGATCATGTAACTCGCCTTGATCGTTGGGAACCGGAGCTGAATGAAGCCATACCAAACGACGAGCGTGACACCACGATGCCTGCAGCAATGGCAACAACGTTGCGCAAACTATTAACTGGCGAACTACTTACTCTAGCTTCCCGGCAACAATTAATAGACTGGATGGAGGCGGATAAAGTTGCAGGACCACTTCTGCGCTCGGCCCTTCCGGCTGGCTGGTTTATTGCTGATAAATCTGGAGCCGGTGAGCGTGGGTCTCGCGGTATCATTGCAGCACTGGGGCCAGATGGTAAGCCCTCCCGTATCGTAGTTATCTACACGACGGGGAGTCAGGCAACTATGGATGAACGAAATAGACAGATCGCTGAGATAGGTGCCTCACTGATTAAGCATTGGTAA

**Tet(M) gene sequence:**

ATGAAAATTATTAATATAGGTGTTTTAGCTCATGTTGACGCAGGAAAAACTACTTTGACAGAAAGCTTACTATATACTAGTGGAGCGATTGCGGAGTCAGGAAGCGTGGATACAGGCACAACAAGAACGGATACTACATTTTTAGAACGTCAGCGAGGAATTACAATTCAGACAGCAGTAACCTCTTTTCAGTGGAAAGATATTAAGGTAAATATCATAGATACTCCAGGACATATGGATTTTTTAGCAGAAGTATATCGCTCGTTATCAGTTTTAGATGGGGCAATCCTACTAATTTCTGCGAGAGATGGAGTACAAGCACAAACTCGGATATTATTTCATGCACTAAATAAAATGGGTATTCCCACAATCTTTTTTATCAATAAGATTGACCAAAATGGGATTGATTTATCAACGGTTTATCAAGATATTAAAGAGAAACTTTCTATGGAAATTATAATCAAACAGAAAGTAGAGCTGCACCCTAATATGTGTGTGATGAGCTGTACGGAACCTGAGCAATGGGATGTGGTAATAGAAGGAAATGATTATCTTTTGGAGAAATATACACTTGGGAAATCATTGGAGATATTAGAACTCGAACAAGAGGAAATCAGAAGATTTCAGAATTGCTCCTTGTACCCTGTTTATCATGGAAGCGCAAAAAGCAACATAGGGATTGAGCAGCTTATAGAAGTGATAACGAATAAATTTTATTCATCAACATACAGAAAGAAGTCTGAACTTTGCGGAAATGTCTTCAAAATTGAATATTCGGAAGAAAGACAACGTCTTGCATATGTACGCCTTTATGGCGGAATCCTGCATTTGCGGGATTCGGTTAGAATATCGGAAAAGGAAAAAATAAAAATTACAGAAATGTATACTTCAATAAATGGTGAATTATGTAAAATTGATAAGGCTTATTCCGGGGAAATTGTTATTTTGCAAAATGAGTTTTTGAAGCTAAATAGTGTTCTTGGAGATACAAAGCTATTGCCACAGAGAGAGAGAATTGAAAATCCGCTCCCTCTGCTGCAAACAACTGTTGAACCGAGCAAACCTCAACAAAGGGAAATGTTACTTGATGCACTTTTAGAAATCTCCGACAGTGACCCGCTTCTACAATATTATGTGGATTCTACGACACATGAAATCATACTTTCTTTCTTAGGGAAAGTACAAATGGAAGTGACTTGTGCTCTATTGCAAGAAAAGTATCATGTGGAGGTAAAAATAAAAAAGCCTACAGTCATTTATATGGAAAGACCGTTAAAAAAAGCAGAGTATACCATTCACATCGAAGTGCCACCGAATCCCTTCTGGGCTTCCATTGGTCTTTCTGTAGCACCGCTTCCATTAGGGAGCGGAGTACAGTATGAGAGCTCGGTTTCTCTTGGATACTTAAATCAATCGTTTCAAAATGCAGTTATGGAAGGGATACGATATGGCTGTGAACAAGGATTGTATGGTTGGAATGTGACGGACTGTAAAATCTGTTTTAAGTATGGCTTATACTATAGCCCTGTTAGTACCCCAGCAGATTTTCGGATGCTTGCTCCTATTGTATTGGAACAAGTTTTAAAAAAAGCTGGAACAGAATTGTTAGAGCCATATCTTAGTTTTAAAATTTATGCACCACAAGAATATCTTTCACGAGCATATAACGATGCTCCTAAATATTGTGCGAACATCGTAGACACTCAACTGAAAAATAATGAGGTCATTCTTAGTGGAGAAATTCCTGCTCGGTGTATTCAAGAATATCGTAATGATTTAACTTTCTTTACAAATGGACGTAGCGTTTGTTTAACAGAGTTAAAAGGGTACTATGTTACTACTGGTGAATCTGTTTGTCAGCCCCGTCGTCCAAATAGTCGGATAGATAAAGTACGATATATGTTCAATAAAATAACTTAG

**nifJp Sequence:**

AAAATGGAAAGAAGCGCTGTTTTAAGCGTGAAGTGAAAAGAATGGCGCTGAAAACCTGTTCAAAACGGCGAAGAGACGTCAAAATTTGTCCAAAAAATACCCGAAATCAAAATTTTACCTGTCAAATTTTTAACAGAGATTTCTGTACGAAAAATAGGACAAAAAATTATCCGAAAAGGTGTGTATACACCCTAATTGAATTGTGTTATAATCGTTAAAGGTAAAAATTTGTTATACACAATTAATACATTTTTAAATGCTTAATTTCTAAGGAGGAATATC

**P2TetO1 Sequence:** repressor=yellow, spacer=blue, TetO1 operator=green

TTAAGACCCACTTTCACATTTAAGTTGTTTTTCTAATCCGCATATGATCAATTCAAGGCCGAATAAGAAGGCTGGCTCTGCACCTTGGTGATCAAATAATTCGATAGCTTGTCGTAATAATGGCGGCATACTATCAGTAGTAGGTGTTTCCCTTTCTTCTTTAGCGACTTGATGCTCTTGATCTTCCAATACGCAACCTAAAGTAAAATGCCCCACAGCGCTGAGTGCATATAATGCATTCTCTAGTGAAAAACCTTGTTGGCATAAAAAGGCTAATTGATTTTCGAGAGTTTCATACTGTTTTTCTGTAGGCCGTGTACCTAAATGTACTTTTGCTCCATCGCGATGACTTAGTAAAGCACATCTAAAACTTTTAGCGTTATTACGTAAAAAATCTTGCCAGCTTTCCCCTTCTAAAGGGCAAAAGTGAGTATGGTGCCTATCTAACATCTCAATGGCTAAGGCGTCGAGCAAAGCCCGCTTATTTTTTACATGCCAATACAATGTAGGCTGCTCTACACCTAGCTTCTGGGCGAGTTTACGGGTTGTTAAACCTTCGATTCCGACCTCATTAAGCAGCTCTAATGCGCTGTTAATCACTTTACTTTTATCTAATCTAGTCATCATTCTAACTAACCTCCTAACAACTTAATTATACCCACTATTATTATTTTTATCAATATATTTTATTCTCCAGTGTTATATACTATAGGGGACCGGTACTCTATCATTGATAGAGTTTGAAACTCTATCATTGATAGAGTATAATATCTTTGTTCATTAGAGCGATAAACTTGAATTTGAAATAAGGAGGAATATTAAAC

**pThlA Sequence:**

TTTTTAACAAAATATATTGATAAAAATAATAATAGTGGGTATAATTAAGTTGTTAGAGAAAACGTATAAATTAGGGATAAACTATGGAACTTATGAAATAGATTGAAATGGTTTATCTGTTACCCCGTATCAAAATTTAGGAGGTTAGATCTT

**J23119 Sequence:**

TTGACAGCTAGCTCAGTCCTAGGTATAATACTAGT

**Table S1:** Plasmids Used in this Work

| **Name** | **Description** | **Source** |
| --- | --- | --- |
| pCL2 | Clostridial vector plasmid with CatP resistance gene and pIP404 origin of replication. | Leang et.al. 2013 |
| pMTL82254 | Clostridial vector plasmid with ermB resistance gene and repA origin of replication. | Heap et.al. 2009 |
| pMTL83151 | Clostridial vector plasmid with CatP resistance gene and repL origin of replication. | Heap et.al. 2009 |
| pCL2.1 | pCL2 with the CatP cassette replaced with ermB from pMTL82254 | This study |
| pELIM2.2 | pCL2 vector with BgaB expression under nifJp promoter | This study |
| pVpas3 | New vector plasmid with enhanced cloning and *E. limosum* regulatory elements based on pCL2. CatP selection marker. | This study |
| pVpas4 | pVpas3 with ermB selection marker rather than CatP. | This study |
| pVpas5 | pVpas3 with ampR selection marker rather than CatP. | This study |
| pVpas6 | pVpas3 with tet(M) selection marker rather than CatP. | This study |
| pVEL1 | Vector plasmid with Catp | This study |
| pVEL2 | Vector plasmid with ermB | This study |
| pVEL4 | Vector plasmid with Tet(M) | This study |
| pEL2 | CreiLOV promoter test plasmid for nifJp | This study |
| pEL3 | CreiLOV promoter test plasmid for p2TetO1 | This study |
| pEL4 | CreiLOV promoter test plasmid for pThlA | This study |
| pEL5 | CatP promoter test plasmid for J23119 | This study |
| pEL2.1 | CatP promoter test plasmid for nifJp | This study |
| pEL3.1 | CatP promoter test plasmid for p2TetO1 | This study |
| pEL4.1 | CatP promoter test plasmid for pThlA | This study |
| pEL5.1 | CatP promoter test plasmid for J23119 | This study |
| pELIM2.2 | Expression of BgaB | This study |
| pELampR2 | Test plasmid for ampR gene | This study |
| pELtetR2 | Test plasmid for TetL gene | This study |
| pELtetM | Test plasmid for TetM gene | This study |
| pELtetW | Test plasmid for TetW gene | This study |
| pELerm | Control plasmid for antibiotic selection marker tests using known ermB gene | This study |

**Table S2:** Oligos Used in this Work

| **Name** | **Description** | **Sequence** |
| --- | --- | --- |
| oAS45 | qPCR amplification of genomic fragment | TTGTAATAAAACTGGATTCGAAAGG |
| oAS46 | qPCR amplification of genomic fragment | ATGAATCCGGAACTTCTTCCC |
| oPAS95 | qPCR amplification of plasmid fragment | CAGCAAAAGGCCAGGAAC |
| oPAS96 | qPCR amplification of plasmid fragment | TTTTGTGATGCTCGTCAGG |
| oPAS99 | Amplification of MCS for pVpas3 | CTAGAACGATCGAAAATGGAAAGAAGC |
| oPAS100 | Amplification of MCS for pVpas3 | CGACAGATAGCTGAAGAGCC |
| oPAS216 | Anneal oligos to generate J23119 promoter with overhangs for pEL5 | ACTAGTATTATACCTAGGACTGAGCTAGCTGTCAAAGTACTGTTTAAACTCGAGG |
| oPAS217 | Anneal oligos to generate J23119 promoter with overhangs for pEL5 | TTGACAGCTAGCTCAGTCCTAGGTATAATACTAGTatggctggacttagacatac |
| oPAS210 | amplify nifJp for assembly of pEL2.pEL2.1 | CCTCGAGTTTAAACAGTACTAAAATGGAAAGAAGCGCTGTTTTA |
| oPAS211 | amplify nifJp for assembly of pEL2 | gtatgtctaagtccagccatGATATTCCTCCTTAGAAATTAAGCATTTAA |
| oPAS240 | Amplify nifJp for assembly of pEL2.1 | ctatttttaTcaattttttcaaataccatGATATTCCTCCTTAGAAATTAAGCATTTAA |
| oPAS212 | Amplify p2TetO1 for assembly of pEL3.1 | CCTCGAGTTTAAACAGTACTttaagacccactttcacatttaag |
| oPAS241 | Amplify p2TetO1 for assembly of pEL3.1 | ctatttttaTcaattttttcaaataccatgtttaatattcctccttatttcaaattc |
| oPAS242 | Amplify pThlA for assembly of pEL4.1 | caactatttttaTcaattttttcaaataccataagaTctaacctcctaaattttgatac |
| oPAS214 | Amplify pThlA for assembly of pEL4.1 | CCTCGAGTTTAAACAGTACTtttttaacaaaatatattgataaaaataataatagtgggt |
| oPAS243 | Anneal oligos to generate J23119 for pEL5.1 | tcaattttttcaaataccatACTAGTATTATACCTAGGACTGAGCTAGCTGTCAA |
| oPAS255 | Anneal oligos to generate J23119 for pEL5.1 | TTGACAGCTAGCTCAGTCCTAGGTATAATACTAGTatggtatttgaaaaaattga |
| oPAS238 | Amplify catP for assembly of promoter screening constructs | atggtatttgaaaaaattgataaaaatagttgg |
| oPAS239 | Amplify catP for assembly of promoter screening constructs | GTTTTATTTGATGCCTGGCAGTTCCCTttaactatttaTcaattcctgcaattcg |
| oPAS116 | Amplify ampR from pCT5-bac1.8 for pELampR2 | gatagcTCGCGatgagtattcaacatttccgtgtcgc |
| oPAS113 | Amplify ampR from pCT5-bac1.8 for pELampR2 | gaTcagTGTACAttaccaatgcttaaTcagtgaggcac |
| oPAS110 | Amplify Tet(L) for pELtetR2 | gaTcagTGTACAttagaaaTccctttgagaatgtttatatac |
| oPAS117 | Amplify Tet(L) for pELtetR2 | gatagcTCGCGatggttttgaacgtctcattac |
| oPAS130 | Amplify Tet(M) for pELtetM | GATAGCTCGCGATGAAAATTATTAATATAGGTG |
| oPAS131 | Amplify Tet(M) for pELtetM | GATCAGTGTACACTAAGTTATTTTATTGAACATATATCG |
| oPAS132 | Amplify Tet(W) for pELtetW | GATAGCTCGCGATGAAAATAATCAATATTG |
| oPAS133 | Amplify Tet(W) for pELtetW | GATCAGTGTACATTACATTACCTTCTGAAAC |
| oPAS64 | Amplify ermB from pMTL82254 for pELerm | ctaagcgcttattggcctcctttttattaaatttatgttacc |
| oPAS65 | Amplify ermB from pMTL82254 for pELerm | taagctagcggagtttaaacacattccctttagtaac |
| oPAS66 | Amplify ermB from pMTL82254 for pVEL2 | tgagcaagtattgtctatttttaatagttaTc |
| oPAS204 | Amplify aadA for pVEL4 | ggtacaaattcccactaagccagcaaatataatgaccctcttgataac |
| oPAS205 | Amplify aadA for pVEL4 | ACAGCGCTTCTTTCCATTTTgtggtagctcttgaTccg |
| oPAS206 | Amplify nifJp for pVEL4 | gccggaTcaagagctaccacAAAATGGAAAGAAGCGCTGTTT |
| oPAS207 | Amplify nifJp for pVEL4 | CCTATATTAATAATTTTCATGATATTCCTCCTTAGAAATTAAGCATTTAAAAATG |
| oPAS208 | Amplify Tet(M) for pVEL4 | AATTTCTAAGGAGGAATATCATGAAAATTATTAATATAGGTGTTTTAGCTCATG |
| oPAS209 | Amplify Tet(M) for pVEL4 | ccacaatattatattataagCTAAGTTATTTTATTGAACATATATCGTACTTTATC |

**Table S3:** Cultures for CreiLOV promoter assessment

| **Condition** | **Strain** | **Details** |
| --- | --- | --- |
| 1 | WT | No aTc |
| 2 | WT | Add 30 ng/mL final concentration aTc (1.5uL 0.1 mg/mL stock) |
| 3 | pEL1 | No aTc |
| 4 | pEL2 col. 5 | No aTc |
| 5 | pEL3 | No aTc |
| 6 | pEL3 | No aTc |
| 7 | pEL3 | Add 30 ng/mL final concentration aTc (1.5uL 0.1 mg/mL stock) |
| 8 | pEL4 col. 3 | No aTc |
| 9 | pEL4 col. 5 | No aTc |
| 10 | pEL5 | No aTc |

**Table S4:** Cultures for *CatP* promoter assessment

| **Tube** | **Strain** | **Details** |
| --- | --- | --- |
| 1 | WT | No aTc |
| 2 | WT | + aTc |
| 3 | pCL2.1 | No aTc |
| 4 | pEL2.1 | No aTc |
| 5 | pEL3.1 | No aTc |
| 6 | pEL3.1 | + aTc |
| 7 | pEL4.1 | No aTc |
| 8 | pEL5.1 | No aTc |

**Table S5:** Plates for *CatP* promoter assessment

| **Plate** | **Details** |
| --- | --- |
| 1 | RCM |
| 2 | RCM + 25 ug/mL Thi |
| 3 | RCM + 50 ug/mL Thi |
| 4 | RCM + 100 ug/mL Thi |
| 5 | RCM + 250 ug/mL Thi |
| 6 | RCM + 500 ug/mL Thi |
